# Reaction dynamics as the missing puzzle piece: the origin of selectivity in oxazaborolidinium ion-catalysed reactions†

**DOI:** 10.1039/d3sc03009a

**Published:** 2023-10-20

**Authors:** Ching Ching Lam, Jonathan M. Goodman

**Affiliations:** a Yusuf Hamied Department of Chemistry, University of Cambridge Lensfield Road Cambridge CB2 1EW UK jmg11@cam.ac.uk

## Abstract

The selectivity in a group of oxazaborolidinium ion-catalysed reactions between aldehyde and diazo compounds cannot be explained using transition state theory. VRAI-selectivity, developed to predict the outcome of dynamically controlled reactions, can account for both the chemo- and the stereo-selectivity in these reactions, which are controlled by reaction dynamics. Subtle modifications to the substrate or catalyst substituents alter the potential energy surface, leading to changes in predominant reaction pathways and altering the barriers to the major product when reaction dynamics are considered. In addition, this study suggests an explanation for the mysterious inversion of enantioselectivity resulting from the inclusion of an *ortho*^i^PrO group in the catalyst.

## Abstract 2

The selectivity in a group of oxazaborolidinium ion-catalysed reactions between aldehyde and diazo compounds cannot be explained using transition state theory.

## Graphical abstract

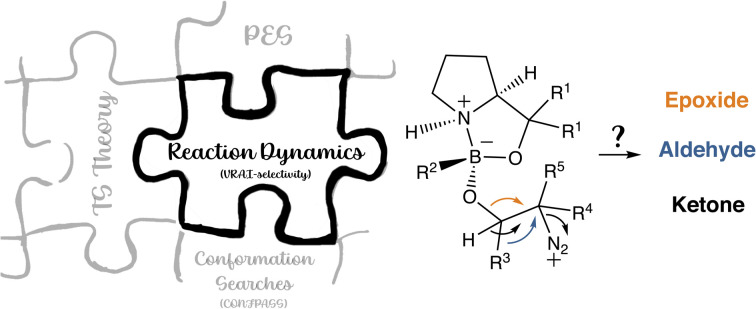

## 1 Introduction

**Fig. 1:**
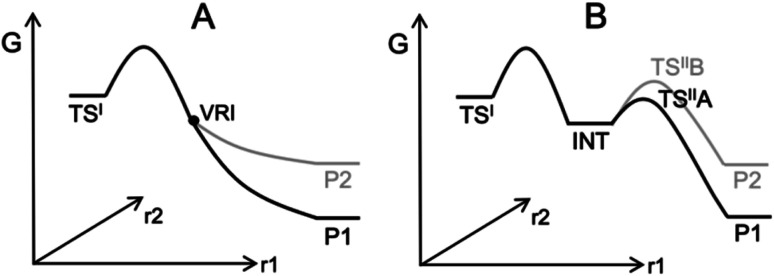
Examples of processes with dynamically controlled selectivity: (A) a bifurcating reaction with a valley-ridge inflection point (VRI) on the potential energy surface^2^ and (B) a reaction with a shadow intermediate where the sequential TSs are low in energy compared to the first TS.^7,8^

Computing organic stereoselectivity is now a standard part of synthetic and mechanistic chemistry.^1^ In general, either the pathway with the lowest energy transition state leads to the preferred product, or else the thermodynamically favourable product is formed. However, there are also reactions where the product selectivity is strongly influenced by reaction dynamics. In these cases, calculation of TS energies by itself is not enough to understand the outcome.^2–8^ Such reactions tend to have bifurcating reaction surfaces^2^ or involve processes with a shallow intermediate that connects two transition states (TS), where the first TS is much higher in energy than the second one (Fig. 1).^7,8^ The role of reaction dynamics has been largely overlooked, with computational organic chemists usually focusing on other factors, such as kinetics, through utilizing the Transition State Theory (TST). One possible reason might be the high computational cost. Traditionally, expensive molecular dynamics (MD) simulations need to be run to produce a large number of reaction trajectories to confirm the effect of reaction dynamics.^9^ Recently, we developed an algorithm, VRAI-selectivity, to analyse and predict selectivity outcomes of dynamically controlled reactions straightforwardly without expensive computations.^10,11^ We developed and tested VRAI-selectivity on about 60 reactions from 25 papers published from 2003 to 2019. Examples include the work on bi- and tri-pericyclic reactions from Houk *et al.*,^12,13^ the study of bifurcating Rh-carbenoid C–H insertions reactions from Tantillo *et al.*^14^ and the publication on dynamic effects in alkene hydroboration from Singleton *et al.*^7^ We surveyed the size of these chemical systems. The number of atoms involved in the reactions ranges from 10 to 50, with a median of 25 (ESI Fig. 7.1†). Recent research has also revealed a growing number of complex organic reactions, in which a significant amount of diligent efforts and computational resources have been deliciated to verify the effect of reaction dynamics. Houk *et al.* have just reported the discovery of tetra-pericyclic cycloadditions.^15^ Ess *et al.* have detailed the dynamic effects in organometallic reactions in their published works.^16^ The research in terpene forming reactions, which are known to be dynamically controlled, is ongoing in the community.^17–19^

**Fig. 2:**
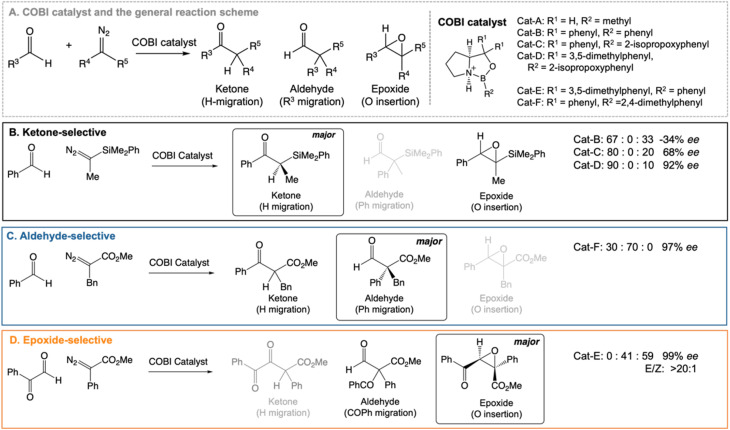
Chiral oxazaborolidinium ion (COBI) catalysed reactions between aldehyde and diazo compounds.^20–22^ We investigated the above reaction systems in this project. All enantiomeric excess (ee) values above are determined from chiral HPLC. For the ketone^22^ and aldehyde^21^-selective reaction, the major to minor product ratio comes directly from the crude product ^1^H NMR. For the epoxide-selective reaction,^20^ the yield for the aldehyde has not been explicitly reported for this reaction. We assume that the loss in percentage yield of the epoxide is all attributed to the aldehyde by-product. The major to minor product ratio is estimated from percentage yield of the isolated *SR* epoxide and diastereomeric ratio, which comes from ^1^H NMR of the crude product. Ryu *et al.* reported that changing the diazo substituent from CO_2_^*t*^Bu to CO_2_Me do not alter the experimental outcome significantly. Thus, we simplified the diazo substituent from CO_2_^*t*^Bu to CO_2_Me for the computational investigation.

Our work highlights the practical relevance of dynamic effects to experimental organic chemistry. We use VRAI-selectivity calculations to investigate a set of complex organic reactions reported by Ryu *et al.*^20–22^ (Fig. 2) and demonstrate that their selectivity is heavily influenced by reaction dynamics. Notably, the chemical systems in this investigation feature significant complexity, encompassing a range of 90–112 atoms. The reactions are catalysed by chiral oxazaborolidinium ion (COBI) catalysts, a well-established and versatile catalyst system developed by Corey *et al.*^23,24^ COBI catalysts effectively activates carbonyl group as a Lewis acid and thus have broad applicability in various organic reactions under research setting.^25–29^ Our study initially centres around the ketone-selective reactions (Fig. 2B).^22^ These reactions involve identical substrates but exhibit distinct chemo- and stereoselective outcomes due to subtle variations in the substituents on the aromatic rings of the catalysts. We show that reaction dynamics plays a key role in determining the selectivity outcome. To validate this finding, we further selected two additional reactions: the epoxide^20^ and aldehyde^21^-selective reaction (Fig. 2C and D), in which the main products are epoxide and aldehyde, respectively. Despite changes in the substituents of diazo and aldehyde substrates, the underlying mechanism remains the same and the reaction outcomes are also governed by reaction dynamics.

We note the publications of previous computational work in understanding the reactivity of COBI catalysts.^30–37^ This has focussed on pericyclic reactions, rationalizing the selectivity based on structural features of the key transition state (TS) structures and the non-covalent interactions between the substrates. To the best of our knowledge, the selected reactions have not been studied considering both reaction dynamics and the full conformational space of the system.

The selectivity controlled by reaction dynamics in complex organic systems is an underexplored area. The findings from this study showcase the efficacy of VRAI-selectivity in filling this gap in our understanding of selectivity and provide more accurate insights into the reaction process. We hope this will open up new avenues for developing better synthesis strategies.

## 2 Computational method

Conformational searching calculations were conducted in MacroModel (V13.4)^38^ with OPLS4 ^39^ force field. DFT calculations were conducted with Gaussian 16 (Revision A.03).^40^ All optimisations were performed at the B3LYP-D3/6-31G(d) level of theory.^41–44^ Single-point energy calculations were carried out at the ωB97X-D/6-311G(d,p) level of theory.^45^ 3D images of the optimised structures were generated with CYLview20.^46^

We have explored the conformational space of key ground state structures thoroughly. CONFPASS was used to assist the DFT re-optimizations of force field structures with confidence that key stable structures are obtained at a reduced computational cost (https://github.com/Goodman-lab/CONFPASS).^47,48^ The default setting was used for generating the priority list for re-optimizing conformers. On average, we are more than 87% confident that the global minimum structure has been obtained after re-optimizing less than 37% of the conformers from the conformational searching output file (ESI Section 1.D†).

**Fig. 3:**
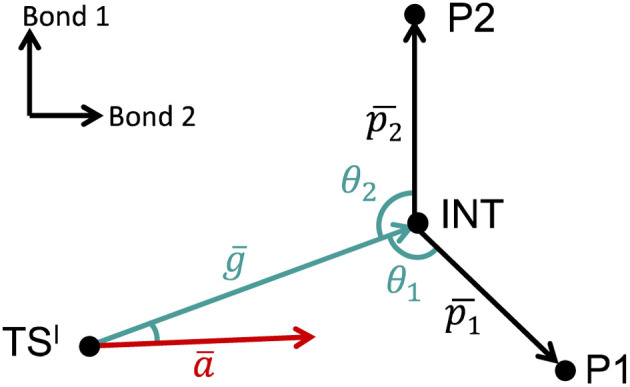
A qualitative illustration of the 2D PES projection from the VRAI-selectivity calculation. *ā* is the imaginary eigenvector of TS^I^. *ḡ* is the separation between TS^I^ and INT. 
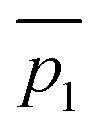
 and 
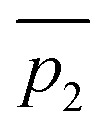
 are the displacement vectors from INT to P1 and P2, respectively.^11^

The VRAI-selectivity algorithm^11^ was utilized to account for the effects of reaction dynamics on reaction selectivity. Before running the main algorithm, an energy check was run at the ωB97XD/6-311g(d,p)//B3LYP-D3/6-31g(d) level of theory to ensure that the second transition states (TS^II^s) are lower in Gibbs free energy than the first transition state (TS^I^). Otherwise, the programme would proceed to calculate the product percentages with the transition state theory (TST). The main VRAI-selectivity algorithm takes the geometries and frequency of the TS^I^, intermediate and products as inputs. The frequency analyses were conducted at the B3LYP-D3/6-31G(d) level of theory in this study. The dimensionality of the potential energy surface (PES) is reduced to two dimensions by examining the bond differences between the products or between a product and TS^I^ based on their geometries (Fig. 3). The major product is determined by the direction in which the imaginary eigenvector of TS^I^ points relative to the intermediate on the 2D PES. The width of the trajectory stream is estimated based on the real eigenvectors of a TS using harmonic oscillator approximation. The selective ratio is predicted by considering the width of the trajectory stream at TS^I^ and how much of it favours each product. In this study, we developed and applied an extension to VRAI-selectivity, VRAI-multi. VRAI-selectivity is designed to be executed on terminals and for dealing with only one set of input files with two products at a time. VRAI-multi automates the process of VRAI-selectivity analyses in situations where there are more than two different products sharing the same intermediate and TS^I^ on their reaction pathways. VRAI-multi is written as a Python package, which allows more flexibility in tuning hyperparameters, such as temperature, and integrations into other Python programmes. The scripts are available at https://github.com/Goodman-lab/VRAI-selectivity.

## 3 Results and discussions

### 3.1 Mechanistic study

**Fig. 4:**
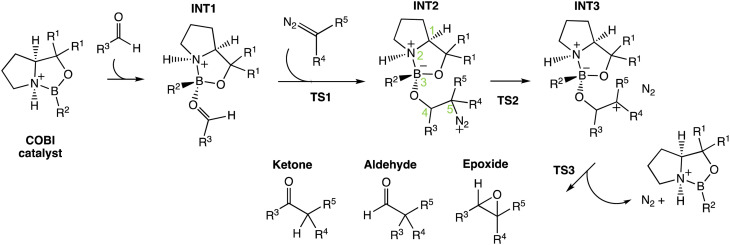
General mechanism for the COBI-catalysed reactions. The key stereo centres in INT2 are numbered.

Previously, Wei *et al.* have conducted a mechanistic study on the chosen aldehyde-selective reaction (Fig. 2C).^37^ We performed a preliminary mechanistic study with a simplified COBI catalyst (*i.e.* Cat-A). The obtained results are applicable to all other reactions and our conclusions align with those of Wei *et al.* (Fig. 2 and 4) The complexation between the COBI catalyst and the aldehyde leads to INT1, where the formed B–O bond is syn to the N–H bond. The Δ*G* of the complexes with the opposite stereochemistry at the boron atom are at least 3.3 kcal mol^−1^ higher compared to INT1 across the various reaction systems in this study (ESI Fig. 4.6†). The addition of the diazo on INT1 gives an adduct, INT2, which undergoes a nitrogen elimination. Sequentially, a 1,2 shift or oxygen insertion occurs to give the products, which can be a ketone, aldehyde or epoxide.

The carbocation structure after the nitrogen elimination, INT3, presents as a shoulder on the PES and the sequential process tends to be barrierless (*i.e.* Δ*G*^‡^(TS3) = 0 kcal mol^−1^). The group involved in the 1,2 shift or oxygen insertion must be at an *anti*-periplanar position relative to the leaving nitrogen. Hence, the stereochemistry of the product is determined by the stereochemistry of INT2 at C4 and C5. The chemoselectivity is controlled by the process after INT2. Thus, the focus of this investigation for understanding selectivity will be on the addition between aldehyde and INT1 *via* TS1 and nitrogen elimination *via* TS2.

There are some exceptions to the trend described above in the epoxide-selective reaction. We will discuss the difference in the following sections.

### 3.2 Predicting selectivity with VRAI-selectivity

We predicted the product percentage for the reaction systems presented in Fig. 2 based on our calculations and compared them to the experimental results.

**Fig. 5:**
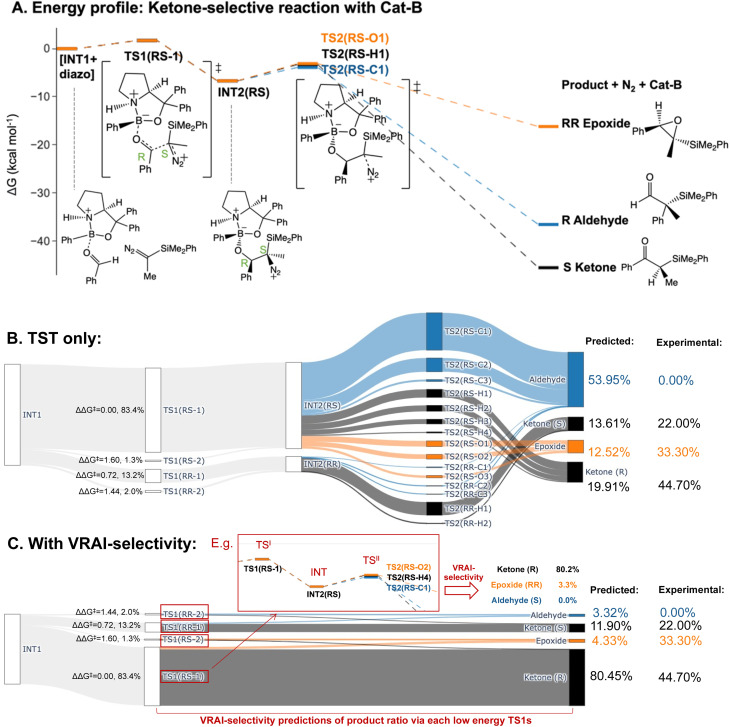
Product percentage calculations for the ketone-selective reaction with Cat-B: (A) the energy profile of the major pathways *via* TS1(*RS*-1), which contribute to 83.4% of the products in the final composition, is presented at the ωB97XD/6-311g(d,p)//B3LYP-D3/6-31g(d) level of theory. The stereochemistry of INT2 at C4 and C5 is *RS*. (B) The Sankey diagram illustrating the percentage calculation of products, based on transition state theory (TST) only, is presented. (C) The predicted product percentages show a better match with the experimental result after incorporating VRAI-selectivity calculations. A VRAI-selectivity calculation is conducted for every pathway *via* a low-energy TS1 (*i.e.* highlighted in red box). The structures on the diagram are named according to the C4 and C5 stereochemistry and numbered based on their energy ranking. For example, TS2(*RS*-C1) is the lowest energy *RS* TS2 that leads to an aldehyde product.

Here, we will use the ketone-selective reaction with Cat-B as an example and elaborate on the procedure for calculating the product percentage. We first considered the transition state theory (TST) and assumed the product percentages are controlled kinetically by TS1s and TS2s. The process of the calculation is illustrated with the Sankey diagram in Fig. 5B. The percentage populations *via* the TSs were computed from the ΔΔ*G*^‡^. Only the low-energy TSs, whose combined population accounted for 99% of the Boltzmann distribution, were selected for the calculation. The final product percentage was determined by summing up the percentage populations of the pathways that contribute to each product. We found distinct mismatches between the experimental and calculated product percentages (Table 1). TST predicts the aldehyde as the major product and contributes to more than 50% of the final product compositions.

**Table 1:** Comparisons to the experimental results. ‘Calculated percentage (TST)’ is derived based on the TST assumption. The selectivity of the reaction entirely depends on the kinetics of TS1 and TS2. ‘Calculated percentage (with VRAI)’ incorporates the VRAI-selectivity calculation outcomes. We assume that the stereochemistry is controlled by kinetics *via* TS1 and the chemoselectivity, *i.e.* processes beyond TS1, are controlled by reaction dynamics

	Experimental percentage	Calculated percentage (TST)	Calculated percentage (with VRAI)
**Ketone-selective – Cat-B**			
Aldehyde	0.00%	53.95%	3.32%
Epoxide	33.30%	12.52%	4.33%
Ketone (*R*)	44.70%	19.91%	80.45%
Ketone (*S*)	22.00%	13.61%	11.90%

**Ketone-selective – Cat-C**			
Aldehyde	0.00%	31.16%	15.41%
Epoxide	25.00%	0.00%	21.41%
Ketone (*R*)	12.00%	0.00%	9.53%
Ketone (*S*)	63.00%	68.84%	53.65%

**Ketone-selective – Cat-D**			
Aldehyde	0.00%	14.34%	11.34%
Epoxide	11.11%	0.00%	6.73%
Ketone (*R*)	3.56%	0.00%	2.80%
Ketone (*S*)	85.33%	85.66%	73.08%

**Epoxide-selective – Cat-E**			
Aldehyde	40.91%	35.80%	44.03%
Epoxide (*SR*)	56.00%	11.90%	38.86%
Epoxide (*RS*)	0.28%	0.00%	0.00%
Epoxide (*SS* + *RR*)	2.81%	52.15%	3.87%
Ketone	0.00%	0.15%	10.32%

**Aldehyde-selective – Cat-F**			
Aldehyde (*S*)	1.04%	0.00%	0.00%
Aldehyde (*R*)	68.95%	8.64%	54.33%
Epoxide	0.00%	0.00%	0.00%
Ketone	30.00%	91.36%	45.67%

We note the large difference between the TST predictions and the experimentally observed selectivity. One possible explanation for this is that the reaction process is partially controlled by reaction dynamics. However, it is also possible that we have missed important transition states. To check the second possibility, we explored the conformational space thoroughly for INT2 using CONFPASS. All possible conformations of TS1 and TS2 were optimized based on the INT2 structures at the DFT level for the ketone-selective reaction with Cat-B. Hence, we are confident that mismatches with the experimental results were not due to insufficient conformation sampling. Secondly, the energy profile (Fig. 5A) reveals features of PES where dynamic effects dominate and control the selectivity. The drop from TS1 to INT2 is more than 8 kcal mol^−1^, while the activation energy for TS2 is less than 3 kcal mol^−1^. This suggests that there is sufficient energy for trajectories to pass over INT2 easily and the process beyond TS1 may be controlled by reaction dynamics. We make this assumption in our analysis and test it by comparison with the experimentally determined outcomes.

We therefore investigated the effects of reaction dynamics using the VRAI-selectivity approach (Fig. 5C). A VRAI-selectivity calculation is performed for every selected low-energy TS1. The analyses were performed with TS1 as the first transition state (TS^I^) and INT2 as the intermediate structure. The product geometries come from the quick reaction coordinate calculations^49^ of the lowest energy TS2 structure that leads to the aldehyde, ketone or epoxide. The percentage population for each pathway was obtained by multiplying the product percentage from VRAI-selectivity analyses and the percentage population of the TS1 from ΔΔ*G*^‡^. By accounting for the effects of reaction dynamics, we successfully predicted the major product chemoselectively and stereoselectively for the reaction with Cat-B. The energy profiles for the ketone-selective reaction with Cat-C and Cat-D are similar to that of the reaction with Cat-B. We repeated the product percentage calculation with and without considering reaction dynamics. The predicted percentages incorporating VRAI-selectivity calculations show much better agreement with the experimental results and suggest that the chemoselectivity is significantly influenced by reaction dynamics.

**Fig. 6:**
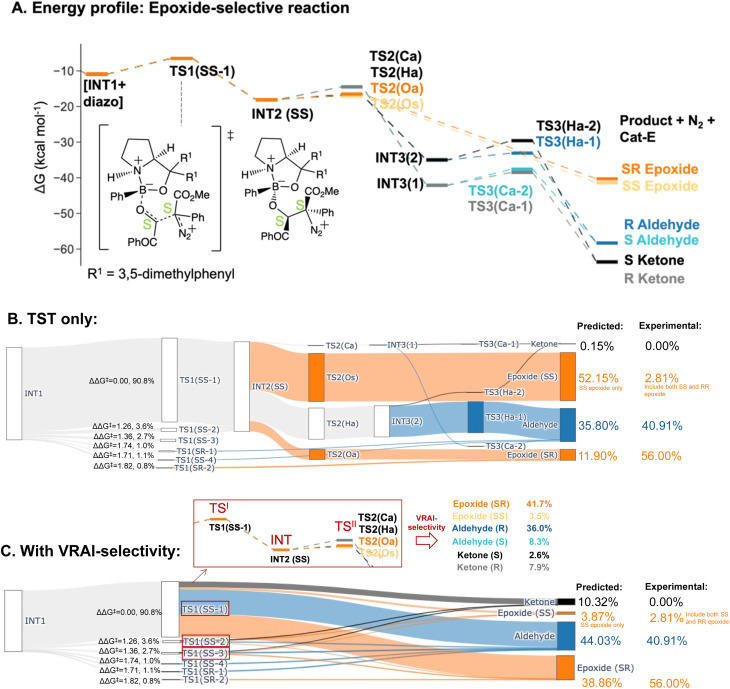
Product percentage calculations for the epoxide-selective reaction with Cat-E: (A) the energy profile of the major pathways *via* TS1(*SS*-1) is presented at the ωB97XD/6-311g(d,p)//B3LYP-D3/6-31g(d) level of theory. The stereochemistry of INT2 at C4 and C5 is *SS*. (B) The Sankey diagram illustrating the percentage calculation of products, based on transition state theory (TST) only, is presented. (C) The predicted product percentages show a better match with the experimental result after incorporating VRAI-selectivity calculations. Three sets of VRAI-selectivity calculations were conducted with TS1(*SS*-1), TS1(*SS*-2) and TS1(*SS*-3) as the first transition state (*i.e.* highlighted in red box in the Sankey diagram). The structures on the diagram are named according to the C4 and C5 stereochemistry position and numbered based on their energy ranking. Experimentally, there are also 0.28% of the *RS* epoxide in the final composition, which is omitted in the above diagram. The calculations, with and without VRAI-selectivity, predict that no *RS* epoxide should be obtained.

The PES of the epoxide-selective reaction is noticeable different from the ketone-selective reactions (Fig. 6A and B). Firstly, in minor pathways *via* TS1(*SS*-4), TS1(*SR*-1) and TS1(*SR*-2), the process beyond TS1 is barrierless and leads to the final product directly. Secondly, in the major pathway (*i.e. via* TS1(*SS*-1)), the *syn*-periplanar insertion (*i.e.* the inserting shifting group at the *syn*-periplanar position to the leaving nitrogen) has comparable kinetic barrier to the *anti*-periplanar insertion. The TS1 structure with the lowest Δ*G*^‡^, TS1(*SS*-1), contributes to both *SR* and *SS* epoxide. Unlike in other reactions, INT3 structures presents as a stable intermediate in the energy profile (Δ*G*^‡^(TS3) > 0 kcal mol^−1^) and can lead to both ketone (*R*/*S*) and aldehyde (*R*/*S*) upon 1,2-shift of the key group. Thus, all possible six products were considered in the VRAI-selectivity calculations for this reaction. The product percentages were calculated with and without incorporating VRAI-selectivity results. The predicted percentages with VRAI-selectivity calculations show a much closer match with the experimental result. The same conclusion as for the ketone-selective reaction can be drawn. The stereoselectivity is controlled kinetically by TS1, while the chemoselectivity is determined by the reaction dynamics of the process beyond TS1.

**Fig. 7:**
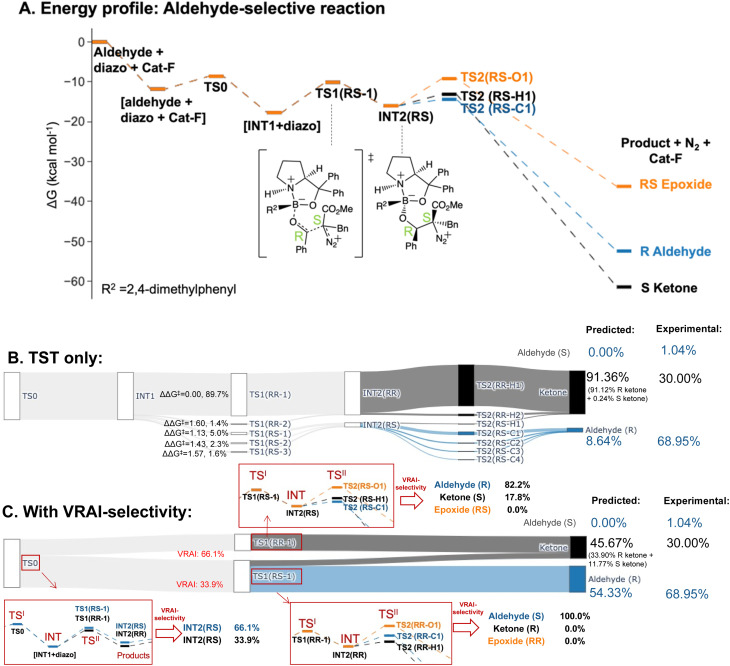
Product percentage calculations for the aldehyde-selective reaction with Cat-F: (A) the energy profile of the major pathways *via* TS1(*RS*-1) is presented at the ωB97XD/6-311g(d,p)//B3LYP-D3/6-31g(d) level of theory. The stereochemistry of INT2 at C4 and C5 is *RS*. (B) The Sankey diagram illustrating the percentage calculation of products, based on transition state theory (TST) only, is presented. (C) The predicted product percentages show a better match with the experimental result after incorporating VRAI-selectivity calculations. Three sets of VRAI-selectivity calculations were conducted to determine the final predicted product percentages. Here, the stereoselectivity is also controlled by reaction dynamics, which differs from other reactions. The structures on the diagram are named according to the C4 and C5 stereochemistry and numbered based on their energy ranking.

The selectivity of the aldehyde-selective reaction was found to be controlled by reaction dynamics entirely. The TS1(*RR*) with the lowest Δ*G*^‡^, with a stereochemistry of *RR* at C4 and C5 position, do not lead to the major product, *R* aldehyde. The earlier study of Wei *et al.*, the selectivity is rationalized based on TST.^37^ Starting from the TS1 with the lowest Δ*G*^‡^ from their publication, which has a *RS* stereochemistry at C4 and C5, we reinvestigated the system, using a functional with a dispersion correction and thorough conformational exploration. This led to a different conclusion from TST which was now incompatible with the experimental result (ESI Table 5.1†). We investigated, therefore, whether the stereochemistry of the reaction may also be controlled by reaction dynamics. The drop in energy from TS0, the TS for the COBI catalyst and aldehyde complexation, to INT1 is more than 10 kcal mol^−1^. TS0 is more than 2.5 kcal mol^−1^ higher compared to the lowest energy TS1 (Fig. 7A). We conducted an additional VRAI-selectivity calculation with TS0 as the TS^I^ and INT1 as the intermediate. The products are stereoisomers of INT2, INT2(*RR*) and INT2(*RS*). As the lowest energy TS2 to the epoxide is higher in energy than the corresponding TS1, this pathway is considered to be unfavourable kinetically. The final product percentages with VRAI-selectivity results were calculated based on three sets of VRAI-selectivity calculations. The Sankey diagram in Fig. 7C provides the details to the calculation. The calculated percentages incorporating VRAI-selectivity calculation results show a better match with the experimental result than the calculated percentages based on TST (Table 1). This implies that both the stereochemistry and chemoselectivity in the aldehyde-selective reaction may be influenced by reaction dynamics.

Inclusion of SMD solvent models^50,51^ in single-point energy calculations and structure re-optimisations with other functionals (*i.e.* ωB97XD, M06-2X and CAM-B3LYP) were considered for key TS1, TS2 and INT2 structures with an ΔΔ*G*^‡^ < 2.5 kcal mol^−1^. We re-calculated the product percentages to reproduce Table 1 (ESI Tables 5.2 and 5.5†). Using a different set of energy did not change result patterns and the same conclusions can be drawn. The mean absolute error (MAE) of the calculated percentages compared to the experimental percentages were considered for various reaction systems at different of theory (ESI Tables 3.11, 5.3 and 5.6†). In all cases, the calculated percentages with VRAI-selectivity have a noticeably lower MAE than those based on TST only.

Quasi-classical MD calculations are regarded as the gold-standard approach to confirm that dynamics effects are important in reaction selectivity. They are much more computationally demanding than the VRAI-selectivity approach. Therefore, we carried out MD simulations with Jprogdyn^52^ on the ketone-selective reaction with Cat-B (see ESI Section 6†). We ran ten trajectories for up to 6 ps from the lowest energy TS, which took a month with our available computer systems. These calculations took four million times more CPU time from the identification of the TS than using VRAI-selectivity. The outcome did not correspond to the experimental result. If we had a hundred times more computer power, we would run more trajectories, starting from a range of low-energy TS. This would probably give an outcome that corresponds to the experimental outcome, but changes a demanding computational problem into an impractical one. VRAI-selectivity introduces some new approximations but gives answers consistent with experimental data almost instantly provided with the DFT calculation results.

In our analysis, we have considered steps to be controlled either by reaction dynamics or by TST. Even if the excess energy is large, however, partial dynamic control can occur, where some of the excess energy is dissipated to the environment and some by internal vibrational energy redistribution. The excess energy for the different processes is given in ESI Table 3.12.† A detailed analysis of the competition between these processes would require more data than we have available, and is a topic for further study.

Overall, the findings demonstrate the importance of considering both reaction kinetics and dynamics in predicting selectivity. A detailed breakdown of the calculations is given in the ESI (ESI Section 3).†

### 3.3 Implications for selectivity

Our results lead to some general implications on explaining the reaction selectivity.

#### 3.3.1 Stereoselectivity

This section focuses on the ketone-selective reactions with Cat-B, Cat-C and Cat-D. The enantioselectivity for these reactions exhibits significant variation with slight modifications in the COBI catalyst, specifically the addition of the oxy-isopropyl (^i^PrO) and methyl groups on the phenyl rings. As previously concluded, the stereochemistry for these systems is governed by TS1.

**Fig. 8:**
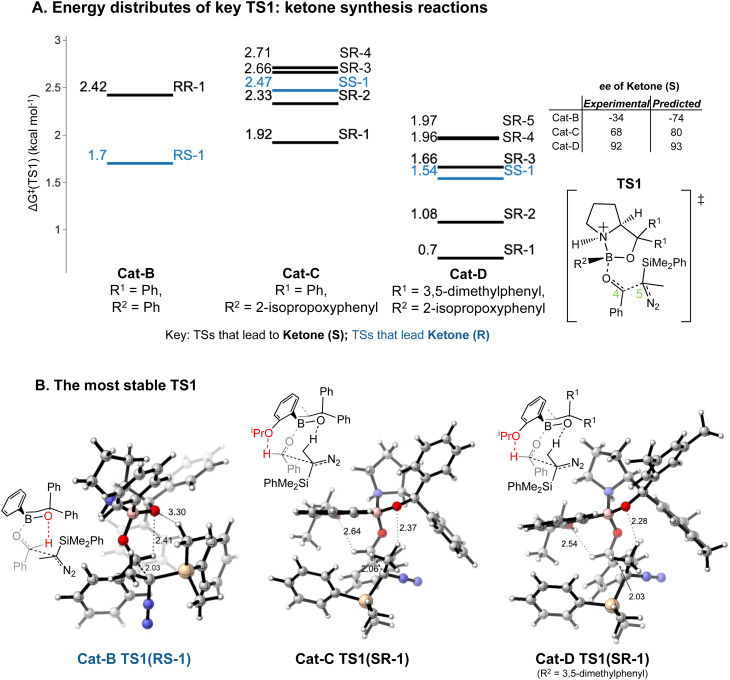
Key TS1s of ketone-selective reactions: (A) an energy level diagram for key TS1s (with ΔΔ*G*^‡^ < 1.4 kcal mol^−1^) is presented at the ωB97XD/6-311g(d,p)//B3LYP-D3/6-31g(d) level of theory. For each TS1, the Δ*G*^‡^ in kcal mol^−1^ and stereochemistry at C4 and C5 centre are labelled. Δ*G*^‡^ values are calculated relative to the lowest energy corresponding INT1 and diazo complex. The TS1 with *SR* and *SS* stereochemistry in reaction with Cat-B and TS1 with *RR* and *RS* stereochemistry in reaction with Cat-C and Cat-D have a ΔΔ*G*^‡^ > 1.4 kcal mol^−1^. (B) The structure of the TS1 with the lowest Δ*G*^‡^: the bond length of key H-bonding interactions and the forming C–C bond are labelled in the diagram. The structures on the diagram are named according to the C4 and C5 stereochemistry and numbered based on their energy ranking. The orientations of both the aldehyde and the diazo are inverted by the possibility of a hydrogen bond to the *ortho*^i^PrO group.

Introducing an ^i^PrO group at position 2 of the phenyl ring attached to boron alters the predominant reaction pathways and reverses the ketone enantioselectivity. For the reaction with Cat-B, the pathways *via* the lowest energy *RR* and *RS* TS1 contribute to 98% of the final product composition. For reactions with Cat-C and Cat-D, TS1 structures with a *SR*/*SS* configuration are lower in Δ*G*^‡^ than *RR*/*RS* TS1 structures. As opposed to Cat-B, reaction systems with Cat-C and Cat-D are more flexible and we obtained multiple stable *SR* TS1 with a ΔΔ*G*^‡^ difference of less than 1.4 kcal mol^−1^ compared to the global minimum (Fig. 8A), which all contribute to the major product of the reaction, *S* ketone. The low-energy *SR* TS1s share similar geometries. The subtle conformational differences include different fused ring geometry and rotation of the ^i^PrO group (ESI Section 4.B†). A critical common feature in these low-energy *SR* TS1 is the stabilising H-bonding interactions between the oxygen from the ^i^PrO group and hydrogen from the aldehyde (Fig. 8B). In the reaction with Cat-B, the critical H-bond is absent in the lowest energy TS1 and the aldehyde adopts the reverse orientation, which leads to the *R* ketone as the major product. This leads to an intuitively-satisfying explanation for the reversal in enantioselectivity arising from a small change in catalyst structure.

**Fig. 9:**
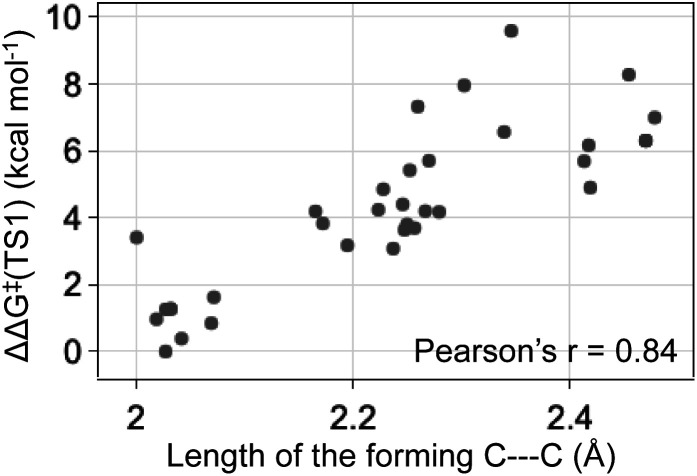
The ketone-selective reaction with Cat-D: ΔΔ*G*^‡^ of TS1 *vs.* the forming C–C bond length. Level of theory: ωB97XD/6-311g(d,p)//B3LYP-D3/6-31g(d).

The energy differences between the key TS1s are due to multiple factors besides the key H bond interactions. We also considered the dispersion interactions and distortions of the structure during the C–C bond formation. Distortion-interaction analyses were carried out on key TS1 structures with Cat-B, Cat-C and Cat-D. Stable TS1 structures tend to exhibit a more pronounced degree of structural distortion compared to the ground state as well as stronger non-covalent interactions between the diazo and INT1 complex. The results echo with the strong positive correlation between the ΔΔ*G*^‡^ of TS1 and the length of the forming C–C bond across reactions with Cat-B, Cat-C and Cat-D (Fig. 9). Low-barrier C–C bond-forming pathways tend to have an earlier TS1.

Reactions with Cat-C and Cat-D are differed by the methyl groups at position 3 and 5 of the R^1^ phenyl rings, which change the energy distributions of key TS1 structures (Fig. 8A). Firstly, Δ*G*^‡^ of the TS1s in the reaction with Cat-D is slightly lower compared to the reaction with Cat-C. Secondly, ΔΔG^‡^ difference between the lowest energy *SR* TS1 and *SS* TS1 increases from using Cat-C to Cat-D, which effectively reduces the percentage of the minor *R* ketone product. Thirdly, VRAI-selectivity analyses on the process beyond TS1 show changes in the PES upon the addition of methyl groups. Across pathways *via* the low-energy *SR* TS1s, the *S* ketone is more dynamically favourable compared to *R* aldehyde and *SS* epoxide in the reaction with Cat-D compared to Cat-C.

#### 3.3.2 Chemoselectivity

This section compares the epoxide and aldehyde-selective reaction with a focus on chemoselectivity. Ryu *et al.* have commented that the electron-withdrawing group substituted aldehyde is in favour of the pathway to epoxide.^20^ Besides the aldehyde substrate, the chosen epoxide and aldehyde-selective reaction only differ by alkyl substituents on the phenyl groups in the catalyst and diazo substrate.

Introducing the electron-withdrawing carbonyl group on the aldehyde substrate changes the PES significantly (Fig. 6A and 7). The pathway to epoxide is kinetically unfavourable in the aldehyde-selective reaction and higher in free energy than the corresponding TS1. TS2 structures to the epoxide have a Δ*G*^‡^ of at least 6.72 kcal mol^−1^, which corresponds to a ΔΔ*G*^‡^ = 5.16 kcal mol^−1^ when compared to the lowest energy TS2 structure. In the epoxide-selective reaction, the Δ*G*^‡^ for TS2 structures to the epoxide are approximately 1 kcal mol^−1^. On the other hand, the corresponding TS2s to aldehyde and ketone have comparable kinetic barriers with a Δ*G*^‡^ in the range of 1.2–3.5 kcal mol^−1^ in both the epoxide and aldehyde-selective reaction. The change in the energy barrier of the epoxide pathways results in the process being dynamically controlled and lead to different chemoselectivities. We conducted Hirshfeld charge analyses and studied the charge distribution by functional groups on key TS structures (ESI Section 4.C†). The differences in charge distribution are insignificant across TS2s from both reactions. However, in TS1(*SS*-1) of the epoxide-selective reaction (Δ*G*^‡^ = 4.43 kcal mol^−1^), the COPh group on the aldehyde withdraws negative charges and is noticeably less positive compared to the corresponding Ph group in the TS1(*RS*-1) of the aldehyde-selective reaction (Δ*G*^‡^ = 7.59 kcal mol^−1^). The chemoselectivity of the epoxide-selective reaction is dynamically controlled by the trajectory from TS1 and the presence of the COPh group affects the charge distribution in TS1.

## 4 Conclusions

Our investigation of the COBI-catalysed reactions suggests that reaction dynamics play a significant role in controlling selectivity. In the ketone and epoxide-selective reactions, the stereochemistry is controlled kinetically by the initial addition between the diazo and the catalyst–aldehyde complex (TS1). However, the process beyond TS1 is controlled by reaction dynamics, which influence the chemoselectivity of the outcome. In the aldehyde-selective reaction, both the stereoselectivity and the chemoselectivity are dynamically controlled.

We also conclude some general implications on the reaction selectivity. Modifying the substituents on the substrate or catalyst leads to changes in the PES. In the ketone-selective reaction systems, the addition of an ^i^PrO group on the catalyst reverses the orientation of the aldehyde by providing the opportunity to for a new, favourable hydrogen bond. This leads to a reverse in enantioselectivity. Changing the phenyl rings to 3,5-dimethylphenyl favours the major product (*i.e. S* ketone) both chemo- and stereo-selectively. COPh-substituted aldehyde lowers the reaction barrier beyond TS1, which favours the synthesis of epoxides.

These results highlight the need for a more thorough explorations of conformational space and demonstrate the effectiveness of the VRAI-selectivity algorithm in predicting outcomes of dynamically controlled processes. Our study provides an important step towards a deeper understanding of the complex interplay between reaction dynamics and selectivity in organic synthesis. Moving forward, we encourage the computational organic chemistry community to consider the procedure presented in this paper, which will provide a more comprehensive and accurate understanding of chemical reactivity to drive the development of better catalyst and synthesis strategies.

## Data availability

All the data for this paper is available on Cambridge Apollo Repository: https://doi.org/10.17863/CAM.96901. CONFPASS and VRAI-selectivity are available to download on GitHub (github.com/Goodman-lab/).

## Author contributions

Prof. J. M. Goodman conceptualised and supervised the project. C. C. Lam performed the research and wrote the paper with the guidance of Prof. J. M. Goodman.

## Conflicts of interest

There are no conflicts of interest.

## Supplementary Material

SC-014-D3SC03009A-s001
