# Supplementary material for: Reaction dynamics as the missing puzzle piece: the origin of selectivity in oxazaborolidinium ion-catalysed reactions
Source: Chem Sci. 2023 Oct 20;14(43):12355–65. doi: 10.1039/d3sc03009a (PMC10631253; doi:10.1039/d3sc03009a)
Supplement: SC-014-D3SC03009A-s001 [file SC-014-D3SC03009A-s001.pdf]

Electronic Supplementary Material (ESI) for Chemical Science.
This journal is © The Royal Society of Chemistry 2023

Supporting Information:

Reaction dynamics as the missing puzzle piece: the origin of selectivity in oxazaborolidinium ion-catalysed reactions

Ching Ching Lam and Jonathan M. Goodman

Table of Contents

1. Computational methodologies 3
2. The uncatalysed pathway 7
3. Calculation breakdowns 8
4. Explaining selectivity 20
5. Benchmarking 26
6. Quasi-classical molecular dynamics 30
7. Notes on pervious work 32
8. Key Structures 33
9. Reference 38

1. Computational methodologies

A. Density functional theory (DFT) calculations

The optimised structures obtained from DFT calculations were validated through frequency analyses, confirming that they correspond to either a minimum or a first-order saddle point on the potential energy surface (PES). Quick reaction coordinate (QRC) calculations[1] were conducted to further verify that the identified TSs correspond to the relevant processes of interest.

B. Conformational searching calculations

Conformational searching calculations were conducted in MacroModel (v13.4) with MacroModel (release 2021-4).[2] The OPLS4[3] force field was employed with the mixed torsional/low-mode sampling method and a maximum of 2000 steps as the limit. Conformers with energies within a window of 41 kJ mol^{-1} (equivalent to 10 kcal mol^{-1}) were saved for subsequent analyses.

C. Data analyses and scripts

Data analyses, such as processing outputs from DFT calculations, were conducted with Python (3.8.12). Graphs were created with Matplotlib (3.3.2) or Plotly (5.1.0)[4].

D. The use of CONFPASS

CONPFASS (https://github.com/Goodman-lab/CONFPASS)[5] was used to assist in re-optimisations of force field structures at the DFT level with confidence that key stable structures are obtained. The default setting (*pipeline-mix*, x = 0.8 and Q = 0.2) was used for generating the priority list for re-optimising conformers. We have ensured that the %Conf at r_{opt} is greater than 80% before terminating the re-optimisation process. r_{opt} is the number of re-optimised conformers over the total number of conformers in the conformational searching output file. %Conf refers to the confidence that the re-optimisation process can be terminated.

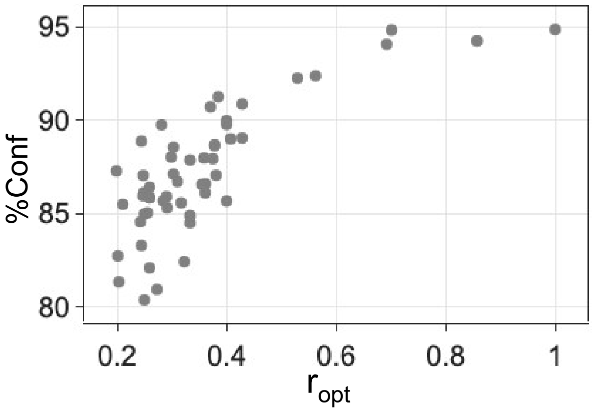

SI Figure 1.1. Distribution of the final %Conf and r_{opt} from the CONFPASS test for confirming the completion of the re-optimisation process (*ie* the global minimum has been obtained).

E. VRAI-selectivity extension: VRAI-multi

VRAI-multi.py is an extension to the VRAI-selectivity.py script (https://github.com/Goodman-lab/VRAI-selectivity)[6,7]. VRAI-multi automates the process of VRAI-selectivity analyses for treating systems with complex PES, *ie* with more than two products that share the same intermediate structure on their reaction pathway.

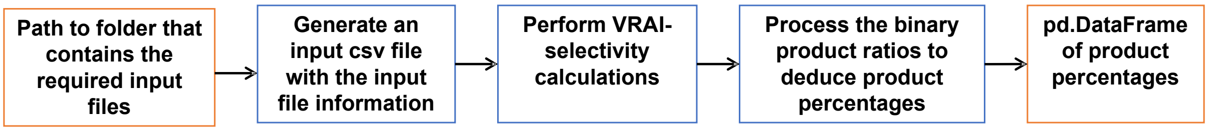

Figure 1.2 The process flow chart for VRAI-multi.py

The input to VRAI-multi is the path to the folder that contains the required files for the VRAI-selectivity analyses. For the script to correctly identify and process the files, the following file naming formats must be adopted:

- Geometry of the first TS in mol file must have a 'TS1.mol' suffix.
- Gaussian16 frequency calculation output file for the first TS must adopt the same filename as the corresponding mol file but with a '.out' suffix.
    - Example: *ol17ryu_e_int2_SSR_11_TS1.mol* and *ol17ryu_e_int2_SSR_11_TS1.out*

- Geometry of the intermediate in .mol file must have a 'int.mol' suffix.
- Gaussian16 frequency calculation output file for the intermediate must adopt the same filename as the corresponding mol file but with a '.out' suffix.
    - Example: *ol17ryu_e_int2_SSR_6_int.mol* and *ol17ryu_e_int2_SSR_6_int.out*

- Gaussian16 frequency calculation output files for the second TS must have a 'TS2.out' suffix. *(optional)*
- Geometry of the product in .mol file must adopt the same filename as the corresponding TS2 with an additional 'prod.mol' as the suffix.
    - Example: there are three different possible products via pathways that share a common intermediate
      ol17ryu_e_int2_SSR_C_1_TS2_prod.mol
      ol17ryu_e_int2_SSR_C_1_TS2.out
      ol17ryu_e_int2_SSR_H_2_TS2_prod.mol
      ol17ryu_e_int2_SSR_H_2_TS2.out
      ol17ryu_e_int2_SSR_O_1_TS2_prod.mol
      ol17ryu_e_int2_SSR_O_1_TS2.out
- Single point energy calculation files must adopt the same filename as the corresponding TS1, int and TS2 structures but with a '_spe.out' suffix. *(optional)*
    - Example: *ol17ryu_e_int2_SSR_O_1_TS2_spe.out* for *ol17ryu_e_int2_SSR_O_1_TS2.out*

The designated folder must contain a minimum of one set of TS1 files, one set of INT files and two product mol files. It is acceptable to include additional sets of product or TS1 files, but the number of intermediate files in the folder should not exceed one set (*ie* a mol and out file).

VRAI-multi generates a data frame that comprises rows containing input file information for conducting VRAI-selectivity calculations. The data frame is constructed to encompass all possible binary combinations of products and TS1s.

Following each row in the input file data frame, the VRAI-selectivity calculations are executed. Binary product ratios are obtained and recorded into a raw result data frame. The raw result table is processed to calculate the product percentages. For results that involve the same intermediate and TS1:

1. The calculation results that share a common product are identified.
2. The product percentages are determined by calculating the ratio of the common product in different sets of results.
3. The above process is repeated for all possible products.

The output of the pipeline is a data frame that contains the product percentages, which are calculated by using different products as the reference in the calculation. It is crucial to verify the consistency of the product percentages obtained from different sets of results to accept them as reliable outcomes.

2. The uncatalysed pathway

The uncatalysed pathways were studied using substrates from the ketone-selective reaction. We simplified the substrates by replacing phenyl with methyl groups. The mechanism for the uncatalysed pathway is distinctively different from the COBI-catalysed pathway. The C-C formation leads to cyclic intermediate structures. The pathway via the 5-membered ring intermediate is more favourable kinetically compared to the pathway via the 4-membered ring intermediate.

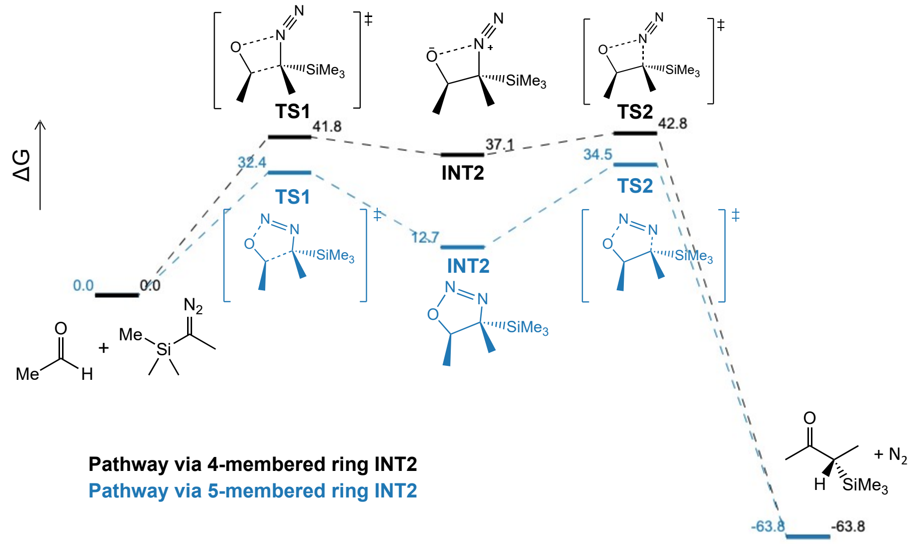

Figure 2.1. The energy profile of the uncatalysed pathway. The ΔG values relative to the reactant are labelled on the diagram in kcal mol^{-1}. The stereochemistry in INT2 is *RR*.

3. Calculation breakdowns

Notes on the result presentations: Unless otherwise specified, the following notations apply to all the tables below.

- Result tables for 'TST only' predictions: The selectivity is controlled kinetically by TS1 and TS2. $\Delta\Delta G^{\ddagger}$ is in kcal mol^{-1} at the ωB97XD/6-311g(d,p)//B3LYP-D3/6-31g(d) level of theory. %(TS1) and %(TS2) corresponds to the percentage population of the TS1 and TS2. %(Pathway) values were calculated as a product of %(TS1) and %(TS2).

- Result tables for 'with VRAI' predictions: %(TS1) refers to the percentage population of the TS1. %(VRAI) is the predicted product percentage from VRAI-selectivity calculations. The frequency analyses required for VRAI-selectivity calculations were conducted at the B3LYP-D3/6-31G(d) level of theory. In the VRAI-selectivity calculation, the given TS1 corresponds to the first transition state and the product structures come from the QRC calculations of the given TS2. %(Pathway) values were calculated as a product of %(TS1) and %(VRAI).

RMSD calculations

Root-mean-square deviation (RMSD) calculations were performed with the GetBestRMS function under the rdkit.Chem.rdMolAlign module on optimised TSs to identify the duplicate structures, which should also be also degenerate in energy. The pair of conformers were considered as having the same structure if the RMSD value < 0.005 Å. The duplicate structures were removed from the data set before calculating product percentages.

The ketone-selective reaction with Cat-B

TST only:

Table 3.1. Calculation breakdowns for the predicted product percentages of the ketone-selective reaction with Cat-B based on TST only. Adding 'ol17ryu_b_int2_S' to labels given in TS1 and TS2 column gives the Gaussian 16 output filename of the structure.

TS1	%(TS1)	ΔΔG‡(TS1)	TS2	%(TS2)	ΔΔG‡(TS2)	%(Pathway)	Product
RR_2_TS1	13.2%	0.72	RR_C_1_TSe	4.9%	1.11	0.7%	R aldehyde
RR_2_TS1	13.2%	0.72	RR_C_2_TSe	3.2%	1.27	0.4%	R aldehyde
RR_2_TS1	13.2%	0.72	RR_C_3_TSe	2.4%	1.39	0.3%	R aldehyde
RR_2_TS1	13.2%	0.72	RR_H_1_TSe	85.8%	0.00	11.3%	S ketone
RR_2_TS1	13.2%	0.72	RR_H_2_TSe	3.7%	1.22	0.5%	S ketone
RR_8_TS1	2.0%	1.44	RR_C_1_TSe	4.9%	1.11	0.1%	R aldehyde
RR_8_TS1	2.0%	1.44	RR_C_2_TSe	3.2%	1.27	0.1%	R aldehyde
RR_8_TS1	2.0%	1.44	RR_C_3_TSe	2.4%	1.39	0.0%	R aldehyde
RR_8_TS1	2.0%	1.44	RR_H_1_TSe	85.8%	0.00	1.7%	S ketone
RR_8_TS1	2.0%	1.44	RR_H_2_TSe	3.7%	1.22	0.1%	S ketone
RS_1_TS1_2	83.4%	0.00	RS_C_1_TSe	43.5%	0.00	36.3%	S aldehyde
RS_1_TS1_2	83.4%	0.00	RS_C_2_TSe	16.0%	0.39	13.3%	S aldehyde
RS_1_TS1_2	83.4%	0.00	RS_C_3_TSe	2.3%	1.15	1.9%	S aldehyde
RS_1_TS1_2	83.4%	0.00	RS_H_1_TSe	6.9%	0.71	5.8%	R ketone
RS_1_TS1_2	83.4%	0.00	RS_H_2_TSe	5.6%	0.80	4.7%	R ketone
RS_1_TS1_2	83.4%	0.00	RS_H_3_TSe	1.4%	1.32	1.2%	R ketone
RS_1_TS1_2	83.4%	0.00	RS_H_4_TSe	9.5%	0.59	8.0%	R ketone
RS_1_TS1_2	83.4%	0.00	RS_O_1_TSe	5.4%	0.81	4.5%	RR epoxide
RS_1_TS1_2	83.4%	0.00	RS_O_2_TSe	6.6%	0.73	5.5%	RR epoxide
RS_1_TS1_2	83.4%	0.00	RS_O_5_TSe	2.8%	1.07	2.3%	RR epoxide
RS_9_TS1	1.3%	1.60	RS_C_1_TSe	43.5%	0.00	0.6%	S aldehyde
RS_9_TS1	1.3%	1.60	RS_C_2_TSe	16.0%	0.39	0.2%	S aldehyde
RS_9_TS1	1.3%	1.60	RS_C_3_TSe	2.3%	1.15	0.0%	S aldehyde
RS_9_TS1	1.3%	1.60	RS_H_1_TSe	6.9%	0.71	0.1%	R ketone
RS_9_TS1	1.3%	1.60	RS_H_2_TSe	5.6%	0.80	0.1%	R ketone
RS_9_TS1	1.3%	1.60	RS_H_3_TSe	1.4%	1.32	0.0%	R ketone
RS_9_TS1	1.3%	1.60	RS_H_4_TSe	9.5%	0.59	0.1%	R ketone
RS_9_TS1	1.3%	1.60	RS_O_1_TSe	5.4%	0.81	0.1%	RR epoxide
RS_9_TS1	1.3%	1.60	RS_O_2_TSe	6.6%	0.73	0.1%	RR epoxide
RS_9_TS1	1.3%	1.60	RS_O_5_TSe	2.8%	1.07	0.0%	RR epoxide

With VRAI:

Table 3.2. Calculation breakdowns for the predicted product percentages of the ketone-selective reaction with Cat-B incorporating VRAI-selectivity calculations (see Figure 5 in the main text for detail). Adding 'ol17ryu_b_int2_S' to labels given in TS1 and TS2 column gives the Gaussian 16 output filename of the relevant structure.

TS1	%(TS1)	TS2	%(VRAI)	%(Pathway)	Product
RR_2_TS1	13.2%	RR_H_1_TSe	89.7%	11.8%	S ketone
RR_2_TS1	13.2%	RR_C_1_TSe	10.3%	1.4%	R aldehyde
RR_8_TS1	2.0%	RR_C_1_TSe	96.1%	2.0%	R aldehyde
RR_8_TS1	2.0%	RR_H_1_TSe	3.9%	0.1%	S ketone
RS_1_TS1_2	83.4%	RS_H_4_TSe	96.1%	80.2%	R ketone
RS_1_TS1_2	83.4%	RS_O_2_TSe	3.9%	3.3%	RR epoxide
RS_1_TS1_2	83.4%	RS_C_1_TSe	0.0%	0.0%	S aldehyde
RS_9_TS1	1.3%	RS_O_2_TSe	80.2%	1.1%	RR epoxide
RS_9_TS1	1.3%	RS_H_4_TSe	19.8%	0.3%	R ketone
RS_9_TS1	1.3%	RS_C_1_TSe	0.0%	0.0%	S aldehyde

The ketone-selective reaction with Cat-C

TST only:

Table 3.3. Calculation breakdowns for the predicted product percentages of the ketone-selective reaction with Cat-C based on TST only. Adding 'ol17ryu_c_int2_S' to labels given in TS1 and TS2 column gives the Gaussian 16 output filename of the structure.

TS1	%(TS1)	ΔΔG‡(TS1)	TS2	%(TS2)	ΔΔG‡(TS2)	%(Pathway)	Product
SR_9_TS1	53.8%	0.00	SR_C_2_TSe	14.8%	0.52	8.0%	R aldehyde
SR_9_TS2	53.8%	0.00	SR_C_3_TSe	5.3%	0.92	2.8%	R aldehyde
SR_9_TS3	53.8%	0.00	SR_C_4_TSe	0.9%	1.60	0.5%	R aldehyde
SR_9_TS4	53.8%	0.00	SR_H_3_TSe	56.2%	0.00	30.2%	S ketone
SR_9_TS5	53.8%	0.00	SR_H_8_TSe	15.0%	0.51	8.1%	S ketone
SR_9_TS6	53.8%	0.00	SR_H_10_TSe	7.3%	0.79	3.9%	S ketone
SR_9_TS7	53.8%	0.00	SR_H_12_TSe	0.6%	1.79	0.3%	S ketone
SR_e3_TS1	18.4%	0.42	SR_C_2_TSe	14.8%	0.52	2.7%	R aldehyde
SR_e3_TS2	18.4%	0.42	SR_C_3_TSe	5.3%	0.92	1.0%	R aldehyde
SR_e3_TS3	18.4%	0.42	SR_C_4_TSe	0.9%	1.60	0.2%	R aldehyde
SR_e3_TS4	18.4%	0.42	SR_H_3_TSe	56.2%	0.00	10.4%	S ketone
SR_e3_TS5	18.4%	0.42	SR_H_8_TSe	15.0%	0.51	2.8%	S ketone
SR_e3_TS6	18.4%	0.42	SR_H_10_TSe	7.3%	0.79	1.3%	S ketone
SR_e3_TS7	18.4%	0.42	SR_H_12_TSe	0.6%	1.79	0.1%	S ketone
SR_e11_TS1	7.9%	0.74	SR_C_2_TSe	14.8%	0.52	1.2%	R aldehyde
SR_e11_TS2	7.9%	0.74	SR_C_3_TSe	5.3%	0.92	0.4%	R aldehyde
SR_e11_TS3	7.9%	0.74	SR_C_4_TSe	0.9%	1.60	0.1%	R aldehyde
SR_e11_TS4	7.9%	0.74	SR_H_3_TSe	56.2%	0.00	4.4%	S ketone
SR_e11_TS5	7.9%	0.74	SR_H_8_TSe	15.0%	0.51	1.2%	S ketone
SR_e11_TS6	7.9%	0.74	SR_H_10_TSe	7.3%	0.79	0.6%	S ketone
SR_e11_TS7	7.9%	0.74	SR_H_12_TSe	0.6%	1.79	0.0%	S ketone
SR_e13_TS1	7.0%	0.79	SR_C_2_TSe	14.8%	0.52	1.0%	R aldehyde
SR_e13_TS1	7.0%	0.79	SR_C_3_TSe	5.3%	0.92	0.4%	R aldehyde
SR_e13_TS1	7.0%	0.79	SR_C_4_TSe	0.9%	1.60	0.1%	R aldehyde
SR_e13_TS1	7.0%	0.79	SR_H_3_TSe	56.2%	0.00	3.9%	S ketone
SR_e13_TS1	7.0%	0.79	SR_H_8_TSe	15.0%	0.51	1.0%	S ketone
SR_e13_TS1	7.0%	0.79	SR_H_10_TSe	7.3%	0.79	0.5%	S ketone
SR_e13_TS1	7.0%	0.79	SR_H_12_TSe	0.6%	1.79	0.0%	S ketone
SS_13_TS1	12.9%	0.56	SS_C_2_TSe	30.1%	0.33	3.9%	S aldehyde
SS_13_TS1	12.9%	0.56	SS_C_3_TSe	69.9%	0.00	9.0%	S aldehyde

With VRAI:

Table 3.4. Calculation breakdowns for the predicted product percentages of the ketone-selective reaction with Cat-C incorporating VRAI-selectivity calculations. Adding 'ol17ryu_c_int2_S' to labels given in TS1 and TS2 column gives the Gaussian 16 output filename of the relevant structure.

TS1	%(TS1)	TS2	%(VRAI)	%(Pathway)	Product
SR_9_TS1	53.8%	SR_H_3_TSe	78.7%	42.4%	S ketone
SR_9_TS1	53.8%	SR_O_8_TSe	21.0%	11.3%	SS epoxide
SR_9_TS1	53.8%	SR_C_2_TSe	0.3%	0.2%	R aldehyde
SR_e3_TS1	18.4%	SR_C_2_TSe	37.6%	6.9%	R aldehyde
SR_e3_TS1	18.4%	SR_H_3_TSe	32.8%	6.0%	S ketone
SR_e3_TS1	18.4%	SR_O_8_TSe	29.7%	5.5%	SS epoxide
SR_e11_TS1	7.9%	SR_H_3_TSe	36.2%	2.9%	S ketone
SR_e11_TS1	7.9%	SR_C_2_TSe	32.9%	2.6%	R aldehyde
SR_e11_TS1	7.9%	SR_O_8_TSe	30.9%	2.4%	SS epoxide
SR_e13_TS1	7.0%	SR_H_3_TSe	34.5%	2.4%	S ketone
SR_e13_TS1	7.0%	SR_C_2_TSe	34.2%	2.4%	R aldehyde
SR_e13_TS1	7.0%	SR_O_8_TSe	31.2%	2.2%	SS epoxide
SS_13_TS1	12.9%	SS_H_1_TSe	74.1%	9.5%	R ketone
SS_13_TS1	12.9%	SS_C_3_TSe	25.9%	3.3%	S aldehyde

The ketone-selective reaction with Cat-D

TST only:

Table 3.5. Calculation breakdowns for the predicted product percentages of the ketone-selective reaction with Cat-D based on TST only. Adding 'ol17ryu_e_int2_S' to labels given in TS1 and TS2 column gives the Gaussian 16 output filename of the structure.

TS1	%(TS1)	ΔΔG‡(TS1)	TS2	%(TS2)	ΔΔG‡(TS2)	%(Pathway)	Product
SR_3_TS1	2.2%	1.27	SR_C_1_TSe	3.7%	1.16	0.1%	R aldehyde
SR_3_TS1	2.2%	1.27	SR_C_2_TSe	3.4%	1.20	0.1%	R aldehyde
SR_3_TS1	2.2%	1.27	SR_C_3_TSe	1.0%	1.67	0.0%	R aldehyde
SR_3_TS1	2.2%	1.27	SR_H_1_TSe	2.2%	1.37	0.0%	S ketone
SR_3_TS1	2.2%	1.27	SR_H_2_TSe	75.4%	0.00	1.7%	S ketone
SR_3_TS1	2.2%	1.27	SR_H_3_TSe	0.8%	1.74	0.0%	S ketone
SR_3_TS1	2.2%	1.27	SR_H_4_TSe	10.0%	0.78	0.2%	S ketone
SR_3_TS1	2.2%	1.27	SR_H_6_TSe	2.1%	1.39	0.0%	S ketone
SR_3_TS1	2.2%	1.27	SR_H_7_TSe	1.3%	1.57	0.0%	S ketone
SR_9_TS1	2.3%	1.26	SR_C_1_TSe	3.7%	1.16	0.1%	R aldehyde
SR_9_TS1	2.3%	1.26	SR_C_2_TSe	3.4%	1.20	0.1%	R aldehyde
SR_9_TS1	2.3%	1.26	SR_C_3_TSe	1.0%	1.67	0.0%	R aldehyde
SR_9_TS1	2.3%	1.26	SR_H_1_TSe	2.2%	1.37	0.1%	S ketone
SR_9_TS1	2.3%	1.26	SR_H_2_TSe	75.4%	0.00	1.7%	S ketone
SR_9_TS1	2.3%	1.26	SR_H_3_TSe	0.8%	1.74	0.0%	S ketone
SR_9_TS1	2.3%	1.26	SR_H_4_TSe	10.0%	0.78	0.2%	S ketone
SR_9_TS1	2.3%	1.26	SR_H_6_TSe	2.1%	1.39	0.0%	S ketone
SR_9_TS1	2.3%	1.26	SR_H_7_TSe	1.3%	1.57	0.0%	S ketone
SR_11_TS1	59.6%	0.00	SR_C_1_TSe	3.7%	1.16	2.2%	R aldehyde
SR_11_TS1	59.6%	0.00	SR_C_2_TSe	3.4%	1.20	2.0%	R aldehyde
SR_11_TS1	59.6%	0.00	SR_C_3_TSe	1.0%	1.67	0.6%	R aldehyde
SR_11_TS1	59.6%	0.00	SR_H_1_TSe	2.2%	1.37	1.3%	S ketone
SR_11_TS1	59.6%	0.00	SR_H_2_TSe	75.4%	0.00	44.9%	S ketone
SR_11_TS1	59.6%	0.00	SR_H_3_TSe	0.8%	1.74	0.5%	S ketone
SR_11_TS1	59.6%	0.00	SR_H_4_TSe	10.0%	0.78	6.0%	S ketone
SR_11_TS1	59.6%	0.00	SR_H_6_TSe	2.1%	1.39	1.3%	S ketone
SR_11_TS1	59.6%	0.00	SR_H_7_TSe	1.3%	1.57	0.8%	S ketone
SR_13_TS1	4.9%	0.97	SR_C_1_TSe	3.7%	1.16	0.2%	R aldehyde
SR_13_TS1	4.9%	0.97	SR_C_2_TSe	3.4%	1.20	0.2%	R aldehyde
SR_13_TS1	4.9%	0.97	SR_C_3_TSe	1.0%	1.67	0.1%	R aldehyde
SR_13_TS1	4.9%	0.97	SR_H_1_TSe	2.2%	1.37	0.1%	S ketone
SR_13_TS1	4.9%	0.97	SR_H_2_TSe	75.4%	0.00	3.7%	S ketone
SR_13_TS1	4.9%	0.97	SR_H_3_TSe	0.8%	1.74	0.0%	S ketone
SR_13_TS1	4.9%	0.97	SR_H_4_TSe	10.0%	0.78	0.5%	S ketone
SR_13_TS1	4.9%	0.97	SR_H_6_TSe	2.1%	1.39	0.1%	S ketone
SR_13_TS1	4.9%	0.97	SR_H_7_TSe	1.3%	1.57	0.1%	S ketone
SR_c9_TS1	22.0%	0.39	SR_C_1_TSe	3.7%	1.16	0.8%	R aldehyde
SR_c9_TS1	22.0%	0.39	SR_C_2_TSe	3.4%	1.20	0.7%	R aldehyde

SR_c9_TS1	22.0%	0.39	SR_C_3_TSe	1.0%	1.67	0.2%	R aldehyde
SR_c9_TS1	22.0%	0.39	SR_H_1_TSe	2.2%	1.37	0.5%	S ketone
SR_c9_TS1	22.0%	0.39	SR_H_2_TSe	75.4%	0.00	16.6%	S ketone
SR_c9_TS1	22.0%	0.39	SR_H_3_TSe	0.8%	1.74	0.2%	S ketone
SR_c9_TS1	22.0%	0.39	SR_H_4_TSe	10.0%	0.78	2.2%	S ketone
SR_c9_TS1	22.0%	0.39	SR_H_6_TSe	2.1%	1.39	0.5%	S ketone
SR_c9_TS1	22.0%	0.39	SR_H_7_TSe	1.3%	1.57	0.3%	S ketone
SR_x1_TS1	2.2%	1.27	SR_C_1_TSe	3.7%	1.16	0.1%	R aldehyde
SR_x1_TS1	2.2%	1.27	SR_C_2_TSe	3.4%	1.20	0.1%	R aldehyde
SR_x1_TS1	2.2%	1.27	SR_C_3_TSe	1.0%	1.67	0.0%	R aldehyde
SR_x1_TS1	2.2%	1.27	SR_H_1_TSe	2.2%	1.37	0.0%	S ketone
SR_x1_TS1	2.2%	1.27	SR_H_2_TSe	75.4%	0.00	1.7%	S ketone
SR_x1_TS1	2.2%	1.27	SR_H_3_TSe	0.8%	1.74	0.0%	S ketone
SR_x1_TS1	2.2%	1.27	SR_H_4_TSe	10.0%	0.78	0.2%	S ketone
SR_x1_TS1	2.2%	1.27	SR_H_6_TSe	2.1%	1.39	0.0%	S ketone
SR_x1_TS1	2.2%	1.27	SR_H_7_TSe	1.3%	1.57	0.0%	S ketone
SS_c13_TS1	6.7%	0.85	SS_C_1_TSe	14.4%	0.58	1.0%	S aldehyde
SS_c13_TS1	6.7%	0.85	SS_C_2_TSe	14.4%	0.58	1.0%	S aldehyde
SS_c13_TS1	6.7%	0.85	SS_C_3_TSe	63.8%	0.00	4.3%	S aldehyde
SS_c13_TS1	6.7%	0.85	SS_C_4_TSe	3.5%	1.13	0.2%	S aldehyde
SS_c13_TS1	6.7%	0.85	SS_C_5_TSe	3.9%	1.09	0.3%	S aldehyde

With VRAI:

Table 3.6. Calculation breakdowns for the predicted product percentages of the ketone-selective reaction with Cat-D based on TST and VRAI-selectivity calculations. Adding ‘ol17ryu_e_int2_S’ to labels given in TS1 and TS2 column gives the Gaussian 16 output filename of the relevant structure.

TS1	%(TS1)	TS2	%(VRAI)	%(Pathway)	Product
SR_3_TS1	2.2%	SR_H_2_TSe	91.2%	2.0%	S ketone
SR_3_TS1	2.2%	SR_O_1_TSe	8.8%	0.2%	SS epoxide
SR_3_TS1	2.2%	SR_C_1_TSe	0.0%	0.0%	R aldehyde
SR_9_TS1	2.3%	SR_H_2_TSe	92.5%	2.1%	S ketone
SR_9_TS1	2.3%	SR_O_1_Tse	7.5%	0.2%	SS epoxide
SR_9_TS1	2.3%	SR_C_1_Tse	0.0%	0.0%	R aldehyde
SR_11_TS1	59.6%	SR_H_2_Tse	88.6%	52.8%	S ketone
SR_11_TS1	59.6%	SR_O_1_Tse	10.2%	6.1%	SS epoxide
SR_11_TS1	59.6%	SR_C_1_Tse	1.3%	0.7%	R aldehyde
SR_13_TS1	4.9%	SR_H_2_Tse	94.2%	4.6%	S ketone
SR_13_TS1	4.9%	SR_O_1_Tse	5.8%	0.3%	SS epoxide
SR_13_TS1	4.9%	SR_C_1_Tse	0.0%	0.0%	R aldehyde
SR_c9_TS1	22.0%	SR_H_2_Tse	43.1%	9.5%	S ketone
SR_c9_TS1	22.0%	SR_C_1_Tse	41.7%	9.2%	R aldehyde
SR_c9_TS1	22.0%	SR_O_1_Tse	15.2%	3.3%	SS epoxide
SR_x1_TS1	2.2%	SR_H_2_Tse	91.2%	2.0%	S ketone

SR_x1_TS1	2.2%	SR_O_1_Tse	8.8%	0.2%	SS epoxide
SR_x1_TS1	2.2%	SR_C_1_Tse	0.0%	0.0%	R aldehyde
SS_c13_TS1	6.7%	SS_H_3_Tse	41.5%	2.8%	R ketone
SS_c13_TS1	6.7%	SS_O_1_Tse	37.6%	2.5%	SR epoxide
SS_c13_TS1	6.7%	SS_C_3_Tse	20.9%	1.4%	S aldehyde

The epoxide-selective reaction

TST:

Table 3.7. Calculation breakdowns for the predicted product percentages of the epoxide-selective reaction based on TST only. %(TS3) corresponds to the percentage population of the TS3. %(Pathway) values are calculated as a product of %(TS1), %(TS2) and %(TS3). Adding 'agw21ryu_d_int2_S' to labels given in TS1, TS2 and TS3 column gives the Gaussian 16 output filename of the structure. NA implies that the process is barrierless and hence no corresponding TS structure is obtained via this pathway.

TS1	%(TS1)	ΔΔG‡(TS1)	TS2	%(TS2)	ΔΔG‡(TS2)	TS3	%(TS3)	ΔΔG‡(TS3)	%(Pathway)	Product
SR_2_TS1	0.8%	1.82	NA	100.0%	NA	NA	100.0%	NA	0.83%	SR epoxide
SR_3_TS1	1.1%	1.71	NA	100.0%	NA	NA	100.0%	NA	1.11%	R aldehyde
SS_10_TS1	2.7%	1.36	SS_Ha_2_TSe	34.8%	0.17	SS_Ha_2_C_TS2_5	99.7%	0.00	0.94%	R aldehyde
SS_10_TS1	2.7%	1.36	SS_Ca_1_TSe	0.1%	2.53	SS_Ca_1_TS2_3	17.9%	0.90	0.00%	S aldehyde
SS_10_TS1	2.7%	1.36	SS_Ca_1_TSe	0.1%	2.53	SS_Ca_1_H_TS2	82.1%	0.00	0.00%	R ketone
SS_10_TS1	2.7%	1.36	SS_Ha_2_TSe	34.8%	0.17	SS_Ha_2_TS2_2	0.3%	3.49	0.00%	S ketone
SS_10_TS1	2.7%	1.36	SS_Oa_2_TSe	11.4%	0.60	NA	100.0%	NA	0.31%	SR epoxide
SS_10_TS1	2.7%	1.36	SS_Ha_3_TSe	53.7%	0.00	NA	100.0%	NA	1.45%	SS epoxide
SS_15_TS1	1.0%	1.74	NA	100.0%	NA	NA	100.0%	NA	1.02%	S aldehyde
SS_50_TS1	3.6%	1.26	SS_Ha_2_TSe	34.8%	0.17	SS_Ha_2_C_TS2_5	99.7%	0.00	1.23%	R aldehyde
SS_50_TS1	3.6%	1.26	SS_Ca_1_TSe	0.1%	2.53	SS_Ca_1_TS2_3	17.9%	0.90	0.00%	S aldehyde
SS_50_TS1	3.6%	1.26	SS_Ca_1_TSe	0.1%	2.53	SS_Ca_1_H_TS2	82.1%	0.00	0.00%	R ketone
SS_50_TS1	3.6%	1.26	SS_Ha_2_TSe	34.8%	0.17	SS_Ha_2_TS2_2	0.3%	3.49	0.00%	S ketone
SS_50_TS1	3.6%	1.26	SS_Oa_2_TSe	11.4%	0.60	NA	100.0%	NA	0.41%	SR epoxide
SS_50_TS1	3.6%	1.26	SS_Ha_3_TSe	53.7%	0.00	NA	100.0%	NA	1.91%	SS epoxide
SS_65_TS1	90.8%	0.00	SS_Ha_2_TSe	34.8%	0.17	SS_Ha_2_C_TS2_5	99.7%	0.00	31.49%	R aldehyde
SS_65_TS1	90.8%	0.00	SS_Ca_1_TSe	0.1%	2.53	SS_Ca_1_TS2_3	17.9%	0.90	0.01%	S aldehyde
SS_65_TS1	90.8%	0.00	SS_Ca_1_TSe	0.1%	2.53	SS_Ca_1_H_TS2	82.1%	0.00	0.06%	R ketone
SS_65_TS1	90.8%	0.00	SS_Ha_2_TSe	34.8%	0.17	SS_Ha_2_TS2_2	0.3%	3.49	0.09%	S ketone
SS_65_TS1	90.8%	0.00	SS_Oa_2_TSe	11.4%	0.60	NA	100.0%	NA	10.35%	SR epoxide

SS_65_TS1	90.8%	0.00	SS_Ha_3_TSe	53.7%	0.00	NA	100.0%	NA	48.79%	SS epoxide

With VRAI:

Table 3.8. Calculation breakdowns for the predicted product percentages of the epoxide-selective reaction based on TST and VRAI-selectivity calculation. Adding 'agw21ryu_d_int2_S' to labels given in TS1 and TS2 column gives the Gaussian 16 output filename of the relevant structure. NA implies that the process is barrierless and hence no corresponding TS structure is obtained via this pathway.

TS1	%(TS1)	TS2/TS3	%(VRAI)	%(Pathway)	Product
SR_2_TS1	0.8%	NA	1.0%	0.0%	SR epoxide
SR_3_TS1	1.1%	NA	1.0%	0.0%	R aldehyde
SS_15_TS1	1.0%	NA	1.0%	0.0%	S aldehyde
SS_10_TS1	2.7%	SS_Ha_2_C_TS2_5	34.3%	0.9%	R aldehyde
SS_10_TS1	2.7%	SS_Ca_1_TS2_3	11.6%	0.3%	S aldehyde
SS_10_TS1	2.7%	SS_Ca_1_H_TS2	8.6%	0.2%	R ketone
SS_10_TS1	2.7%	SS_Ha_2_TS2_2	5.2%	0.1%	S ketone
SS_10_TS1	2.7%	SS_Oa_2_TSe	34.3%	0.9%	SR epoxide
SS_10_TS1	2.7%	SS_Ha_3_TSe	6.0%	0.2%	SS epoxide
SS_50_TS1	3.6%	SS_Ha_2_C_TS2_5	35.6%	1.3%	R aldehyde
SS_50_TS1	3.6%	SS_Ca_1_TS2_3	34.3%	1.2%	S aldehyde
SS_50_TS1	3.6%	SS_Ca_1_H_TS2	2.7%	0.1%	R ketone
SS_50_TS1	3.6%	SS_Ha_2_TS2_2	9.3%	0.3%	S ketone
SS_50_TS1	3.6%	SS_Oa_2_TSe	2.7%	0.1%	SR epoxide
SS_50_TS1	3.6%	SS_Ha_3_TSe	15.4%	0.5%	SS epoxide
SS_65_TS1	90.8%	SS_Ha_2_C_TS2_5	36.0%	32.7%	R aldehyde
SS_65_TS1	90.8%	SS_Ca_1_TS2_3	8.3%	7.6%	S aldehyde
SS_65_TS1	90.8%	SS_Ca_1_H_TS2	7.9%	7.2%	R ketone
SS_65_TS1	90.8%	SS_Ha_2_TS2_2	2.6%	2.3%	S ketone
SS_65_TS1	90.8%	SS_Oa_2_TSe	41.7%	37.8%	SR epoxide
SS_65_TS1	90.8%	SS_Ha_3_TSe	3.5%	3.2%	SS epoxide

The aldehyde-selective reaction

TST only:

Table 3.9. Calculation breakdowns for the predicted product percentages of the aldehyde-selective reaction based on TST only. Adding ‘jacs13ryu2_d_int2_S’ to labels given in TS1 and TS2 column gives the Gaussian 16 output filename of the structure. Some files may have additional suffixes.

Entry	TS1	%(TS1)	ΔΔG‡(TS1)	TS2	%(TS2)	ΔΔG‡(TS2)	%(Pathway)	Product
0	RR_1_TS1_2	89.7%	0.00	RR_H_1_TSe	95.4%	0.00	85.5%	R ketone
1	RR_1_TS1_2	89.7%	0.00	RR_H_3_TSe	4.6%	1.17	4.1%	R ketone
2	RR_3_TS1	1.4%	1.60	RR_H_1_TSe	95.4%	0.00	1.4%	R ketone
3	RR_3_TS1	1.4%	1.60	RR_H_3_TSe	4.6%	1.17	0.1%	R ketone
4	RS_2_TS1	2.3%	1.43	RS_C_1_TSe	73.3%	0.00	1.7%	R aldehyde
5	RS_2_TS1	2.3%	1.43	RS_C_2_TSe	13.0%	0.67	0.3%	R aldehyde
6	RS_2_TS1	2.3%	1.43	RS_C_3_TSe	9.3%	0.80	0.2%	R aldehyde
7	RS_2_TS1	2.3%	1.43	RS_C_4_TSe	1.9%	1.42	0.0%	R aldehyde
8	RS_2_TS1	2.3%	1.43	RS_H_1_TSe	2.6%	1.29	0.1%	S ketone
9	RS_3_TS1	1.6%	1.57	RS_C_1_TSe	73.3%	0.00	1.1%	R aldehyde
10	RS_3_TS1	1.6%	1.57	RS_C_2_TSe	13.0%	0.67	0.2%	R aldehyde
11	RS_3_TS1	1.6%	1.57	RS_C_3_TSe	9.3%	0.80	0.1%	R aldehyde
12	RS_3_TS1	1.6%	1.57	RS_C_4_TSe	1.9%	1.42	0.0%	R aldehyde
13	RS_3_TS1	1.6%	1.57	RS_H_1_TSe	2.6%	1.29	0.0%	S ketone
14	RS_1_TS1	5.0%	1.12	RS_C_1_TSe	73.3%	0.00	3.7%	R aldehyde
15	RS_1_TS1	5.0%	1.12	RS_C_2_TSe	13.0%	0.67	0.7%	R aldehyde
16	RS_1_TS1	5.0%	1.12	RS_C_3_TSe	9.3%	0.80	0.5%	R aldehyde
17	RS_1_TS1	5.0%	1.12	RS_C_4_TSe	1.9%	1.42	0.1%	R aldehyde
18	RS_1_TS1	5.0%	1.12	RS_H_1_TSe	2.6%	1.29	0.1%	S ketone
19	RS_1_TS1	5.0%	1.12	RS_O_1_TSe	0.0%	5.16	0.0%	RS epoxide

With VRAI:

Table 3.10. Calculation breakdowns for the predicted product percentages of the aldehyde-selective reaction based on VRAI-selectivity calculations only. See Figure 7.C in the main text for elaborations on the VRAI-selectivity calculations. %(Pathway) values are calculated as a product of %(VRAI1) and %(VRAI2). Adding ‘jacs13ryu2_d_int2_S’ to labels given in TS0, TS1 and TS2 column gives the Gaussian 16 output filename of the relevant structure. Some files may have additional suffixes.

Entry	TS0	TS1	%(VRAI1)	TS2	%(VRAI2)	%(Pathway)	Product
0	R_1_TS0	RR_1_TS1_2	33.90%	RR_C_1_TSe	0.00%	0.00%	S aldehyde
1	R_1_TS0	RR_1_TS1_2	33.90%	RR_H_1_TSe	100.00%	33.90%	R ketone
2	R_1_TS0	RS_1_TS1	66.10%	RS_C_1_TSe	82.20%	54.33%	R aldehyde
3	R_1_TS0	RS_1_TS1	66.10%	RS_H_1_Tse	17.80%	11.77%	S ketone

Table 3.11. Mean absolute error (MAE) calculations: This table presents the mean absolute error (MAE) data of the calculated percentages compared to the experimental percentages. The level of theory for the calculations is ωB97XD/6-311g(d,p)//B3LYP-D3/6-31g(d).

**MAE compared to the experimental percentage**	
Calculated Percentage (with VRAI)	Calculated Percentage (TST)
Ketone-selective reaction - Cat-B	
19.5%	27.0%
Ketone-selective reaction - Cat-C	
7.7%	18.5%
Ketone-selective reaction - Cat-D	
7.2%	7.3%
Epoxide-selective reaction - Cat-E	
5.4%	19.8%
Aldehyde-selective reaction - Cat-F	
7.8%	30.7%

Table 3.12. ΔG values related to the dynamically controlled steps: ΔG from TS1 to INT2 (ΔG(TS1→INT2)) and ΔG from INT2 to TS2 ($\Delta G^{\ddagger}$(TS2)) of the major pathways are recorded below for each reaction. The major pathway is the pathway with the highest %(Pathway) in the corresponding calculation breakdown table (with VRAI-selectivity) of the reaction. The level of theory is ωB97XD/6-311g(d,p)//B3LYP-D3/6-31g(d).

**ΔG(TS1→INT2)**	**$\Delta G^{\ddagger}$(TS2)**
Ketone-selective reaction - Cat-B	
-8.4	3.4
Ketone-selective reaction - Cat-C	
-9.3	3.2
Ketone-selective reaction - Cat-D	
-9.3	3.9
Epoxide-selective reaction - Cat-E	
-11.6	1.2
Aldehyde-selective reaction - Cat-F	
-7.4	1.6

4. Explaining selectivity

A. Distortion-interaction analyses

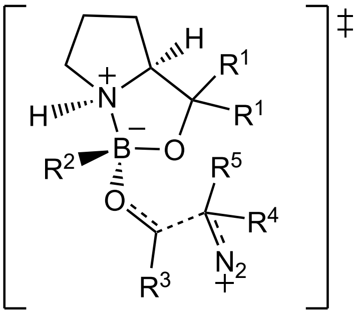

$$\Delta U^{\ddagger} = \Delta U_i^{\ddagger} + \Delta U_d^{\ddagger}$$

$\Delta U^{\ddagger}$: activation energy, U
$\Delta U_i^{\ddagger}$: total interaction energy, U
$\Delta U_d^{\ddagger}$: total distortion energy, U

Figure 4.1. Illustrations for distortion-interaction analyses

We performed distortion-interaction analyses on key TS1 structures for each reaction following the below procedure:

1. The xyz coordinates of the INT1 and diazo compound are extracted from the optimised TS1 structure. We ran single point energy calculations on the distorted INT1 and diazo structure at the ωB97XD/6-311g(d,p) level of theory. The U of the distorted structures were compared to the U of the ground state structures to obtain the distortion energy for INT1 and diazo individually, *ie* the $\Delta U_{d,INT1}^{\ddagger}$ and $\Delta U_{d,diazo}^{\ddagger}$.
2. Deduce $\Delta U_d^{\ddagger}$: $\Delta U_d^{\ddagger} = \Delta U_{d,INT1}^{\ddagger} + \Delta U_{d,diazo}^{\ddagger}$
3. Deduce $\Delta U^{\ddagger}$: $\Delta U^{\ddagger}$ = U(TS1) – (U(ground state INT1)+ U(ground state diazo)). The level of theory for U is ωB97XD/6-311g(d,p).
4. Deduce $\Delta U_i^{\ddagger}$: $\Delta U_i^{\ddagger} = \Delta U^{\ddagger} - \Delta U_d^{\ddagger}$

Table 4.1. Distortion-interaction analysis results. Negative $\Delta U^{\ddagger}$ were obtained as the association between INT1 and diazo was not accounted, which is likely to be very exothermic. Adding the text in the bracket to the TS1 label in each section gives the Gaussian 16 output filename of the relevant structure.

TS1	$\Delta U^{\ddagger}$	$\Delta U_d^{\ddagger}$	$\Delta U_i^{\ddagger}$	$\Delta\Delta U_d^{\ddagger}$	$\Delta\Delta U_i^{\ddagger}$
(ol17ryu_b_int2_S)		**The ketone-selective reaction (Cat-B)**			
RR_2_TS1	33.3	89.2	-55.9	6.7	-10.9
RS_1_TS1_2	33.3	88.5	-55.2	6.0	-10.2
SR_3_TS1	35.9	83.0	-47.1	0.5	-2.1
SS_c13_TS1	37.5	82.5	-45.0	0.0	0.0
(ol17ryu_c_int2_S)		**The ketone-selective reaction (Cat-C)**			
RR_8_TS1	-12.0	32.0	-44.0	3.7	-5.0
RS_5_TS1	-10.6	28.3	-39.0	0.0	0.0
SR_9_TS1	-15.3	38.4	-53.6	10.1	-14.7
SR_e3_TS1	-14.3	39.4	-53.7	11.1	-14.8
SR_e11_TS1	-14.6	39.2	-53.8	10.9	-14.9
SR_e13_TS1	-14.4	39.7	-54.1	11.4	-15.2
SS_13_TS1	-15.8	39.6	-55.3	11.3	-16.4

(ol17ryu_e_int2_S)	**The ketone-selective reaction (Cat-D)**				
RR_10_TS1	-11.8	32.9	-44.7	7.1	-10.3
RS_9_TS1	-8.6	25.8	-34.4	0.0	0.0
SR_c9_TS1	-12.7	41.7	-54.4	15.9	-20.0
SR_3_TS1	-12.9	42.5	-55.4	16.8	-21.0
SR_11_TS1	-13.9	41.5	-55.4	15.8	-21.0
SR_13_TS1	-13.5	41.8	-55.3	16.0	-20.9
SS_c13_TS1	-14.1	41.8	-55.9	16.0	-21.5
(agw21ryu_d_int2_S)	**The epoxide-selective reaction**				
RR_2_11_TS1	-16.3	31.9	-48.2	7.4	-4.2
RS_5_TS1	-18.5	33.0	-51.5	8.6	-7.5
SR_2_TS1	-19.0	30.8	-49.8	6.4	-5.9
SR_3_TS1	-18.5	25.5	-43.9	1.0	0.0
SS_10_TS1	-20.2	33.7	-53.8	9.2	-9.9
SS_15_TS1	-20.0	24.7	-44.7	0.3	-0.8
SS_50_TS1	-20.2	24.4	-44.7	0.0	-0.7
SS_65_TS1	-20.3	27.1	-47.4	2.7	-3.5
(jacs13ryu2_d_int2_S)	**The aldehyde-selective reaction**				
RR_1_TS1_2	-16.6	28.4	-44.9	0.4	-2.9
RR_3_TS1	-14.8	28.8	-43.6	0.8	-1.5
RS_1_TS1	-15.9	28.1	-44.0	0.1	-1.9
RS_2_TS1	-15.9	29.0	-44.9	1.1	-2.8
RS_3_TS1	-15.0	28.0	-43.0	0.0	-1.0
RS_5_TS1	-15.5	28.2	-43.8	0.3	-1.7
SR_2_TS1	-10.9	32.2	-43.1	4.2	-1.0
SS_1_TS1	-11.4	30.7	-42.0	2.7	0.0

B. TS1 structures

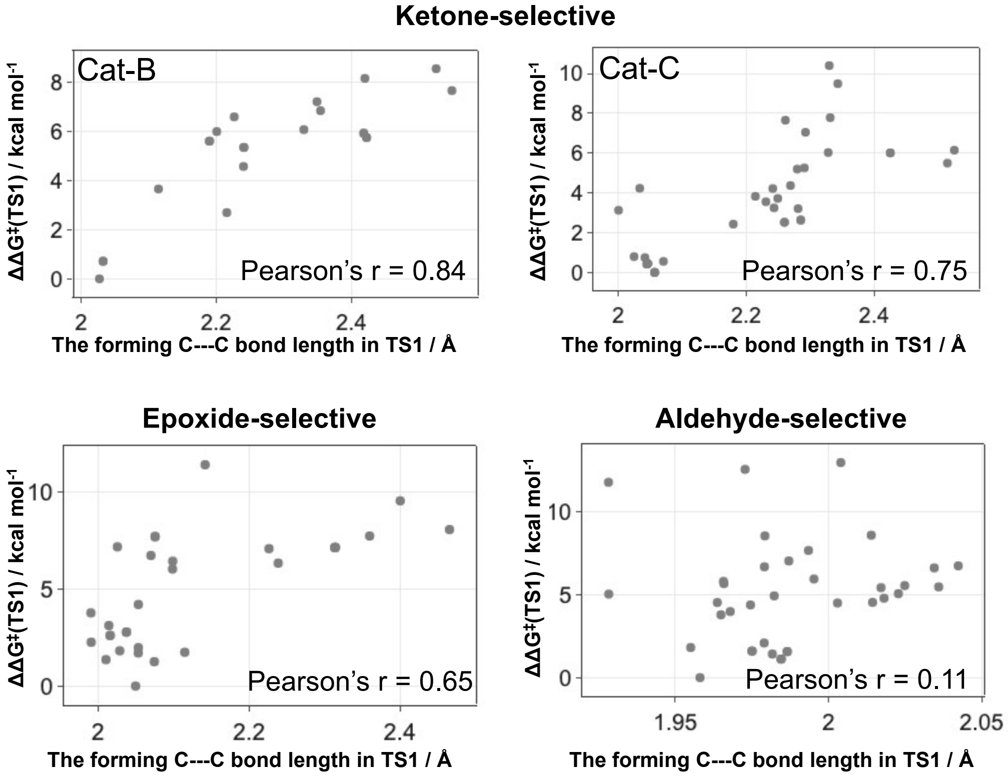

Figure 4.2. ΔΔG‡(TS1) Correlations between ΔΔG‡(TS1) and the bond length of the forming C-C bond. A strong positive correlation is observed in ketone-selective reactions, but not in the case of the aldehyde and epoxide-selective reactions.
Level of theory = ωB97XD/6-311g(d,p)//B3LYP-D3/6-31g(d)

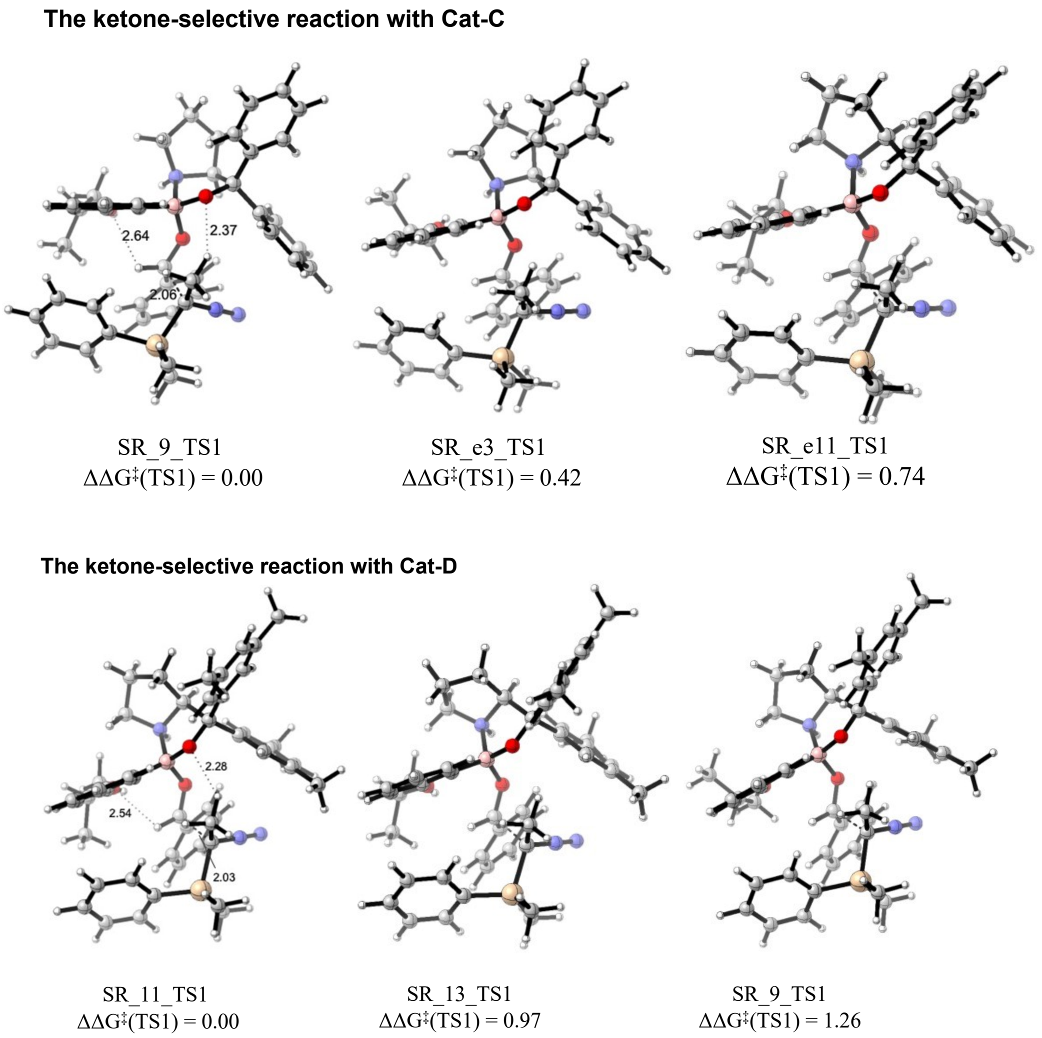

Figure 4.3. Key *SR* TS1 structures in the ketone-selective reaction with Cat-C and Cat-D. These *SR* TS1 shares similar geometries and are all within 1.4 kcal mol^{-1} compared to the global minimum. Level of theory = ωB97XD/6-311g(d,p)//B3LYP-D3/6-31g(d)

C. Charge analyses

Aldehyde-selective: TS1(RS-1)

jacs13ryu2_d_int2_SRS_1_TS1

Group	Charge
N_2	0.347
CO_2Me	0.021
Bn	0.036
Ph	0.101
C5	0.035

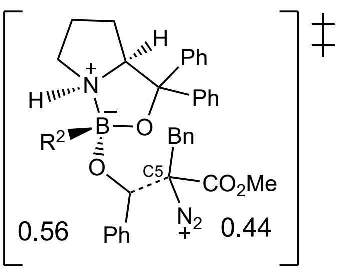

R^2 =2,4-dimethylphenyl

Epoxide-selective: TS1(SS-1)

agw21ryu_d_int2_SSS_65_TS1

Group	Charge
N_2	0.352
CO_2Me	0.054
Ph	0.062
COPh	0.055
C5	0.037

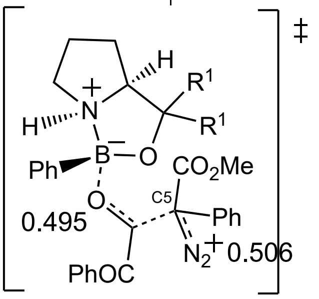

R^1 = 3,5-dimethylphenyl

Ketone-selective with Cat-B: TS1(RS-1)

ol17ryu_b_int2_SRS_1_TS1

Group	Charge
N_2	0.235
$SiMe_2Ph$	0.271
Me	0.052
Ph	0.062
C5	-0.038

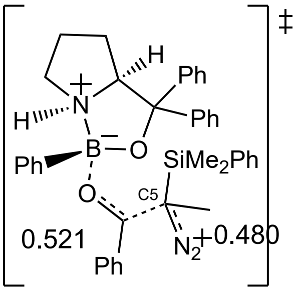

Figure 4.4. Hirshfeld charge analyses on TS1 structures. Pathways that linked with the above TS1 contributes to more 50% of the final product composition.

Aldehyde-selective:

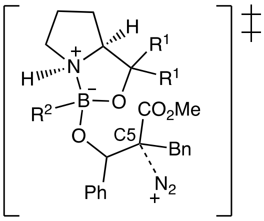

R^1 = phenyl, R^2 =2,4-dimethylphenyl

jacs13ryu2_d_int2_S

	RS_C_1_TSe	RS_H_1_TSe	RS_O_1_TSe
**Product**	**Aldehyde**	**Ketone**	**Epoxide**
N_2	0.327	0.326	0.341
CO_2Me	0.077	0.079	0.080
Bn	0.089	0.146	0.123
Ph	0.066	0.028	0.016
C5	0.157	0.149	0.145

Epoxide-selective:

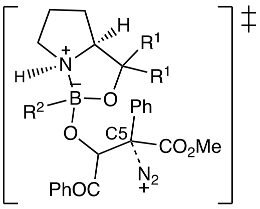

R^1 = 3,5-dimethylphenyl, R^2 = phenyl

agw21ryu_d_int2_S

	SS_Ca_1_TSe	SS_Ha_2_TSe	SS_Oa_2_TSe
**Product**	**Aldehyde**	**Ketone**	**Epoxide**
N_2	0.398	0.311	0.341
CO_2Me	0.072	0.068	0.093
Ph	0.149	0.197	0.170
COPh	0.063	0.113	0.036
C5	0.119	0.123	0.123

Ketone-selective with Cat-B:

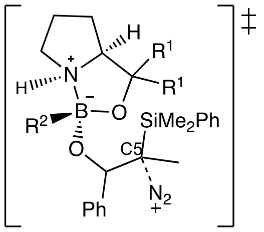

R^1 = phenyl, R^2 = phenyl

ol17ryu_b_int2_S

	RS_C_1_TSe	RS_H_4_TSe	RS_O_2_TSe
**Product**	**Aldehyde**	**Ketone**	**Epoxide**
N_2	0.310	0.320	0.305
$SiMe_2Ph$	0.246	0.275	0.238
Me	0.073	0.080	0.084
Ph	0.067	0.027	0.029
C5	0.084	0.074	0.079

Figure 4.5. Hirshfeld charge analyses on TS2 structures. The chosen TS2 structures have the lowest $\Delta G^{\ddagger}$ among other TS2 structures that lead to the same product.
Level of theory = ωB97XD/6-311g(d,p)//B3LYP-D3/6-31g(d)

D. INT1

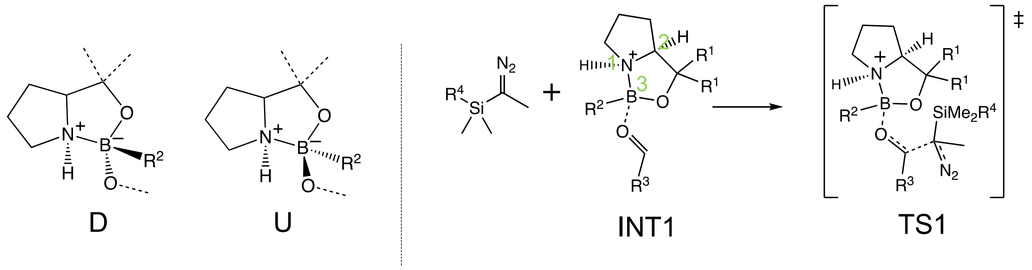

	ΔG(SSD)	ΔG(SSU)
Cat-B	0.0	4.5
Cat-C	0.0	3.7
Cat-D	0.0	3.3

Unit: kcal mol^{-1}

Figure 4.6. The INT1 diastereoisomer stability in ketone-selective reactions: The catalysts have a stereochemistry of *SS*. If the formed B-O bond is syn to the N-H bond, 'D' label is given. The 'U' label refers to the opposite stereochemistry, *ie* formed B-O bond is anti to the N-H bond.
Level of theory = ωB97XD/6-311g(d,p)//B3LYP-D3/6-31g(d)

5. Benchmarking

A. Comparisons with previous studies

Wei *et al.* have previously conducted a mechanistic study on our chosen reactions for the aldehyde-selective reaction at the B3LYP/6-31g(d)/propionitrile/PCM level of theory. We extracted the xyz coordinates of the key TS1 structures from the supporting information of their work. We re-optimised the structures and calculate ΔΔG‡ with the theory level used in this study, *ie* ωB97XD/6-311g(d,p) //B3LYP-D3/6-31g(d).

Table 5.1. Comparisons with previous studies. Adding the text in the bracket to the TS1 label in each section gives the Gaussian 16 output filename of the relevant structure. ΔΔG‡(TS1) values are calculated based on the ΔG‡(TS1) of the jacs13ryu2_d_int2_SRR_1_TS1_2 structure from this work.

TS1	Stereochemistry at C4 and C5	ΔΔG‡(TS1) / kcal mol^{-1}
(jacs13ryu2_d_int2_S)	This work	
RR_1_TS1_2	*RR*	0.00
RS_1_TS1	*RS*	1.12
SR_2_TS1	*SR*	4.54
SS_1_TS1	*SS*	4.80
(jpca15wei_TS2_)	Wei *et al.* after re-optimisation at B3LYP-D3/6-31g(d)	
RR_TS1	*RR*	6.69
RS_TS1	*RS*	1.57
SR_TS1	*SR*	12.58
SS_TS1	*SS*	8.56

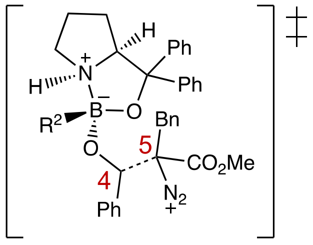

R^2 = 2,4-dimethylphenyl

B. Solvent Model

Inclusion of solvent models[8,9] in single-point energy calculations were considered for key TS1 and TS2 structures with an $\Delta\Delta G^{\ddagger} < 2.5$ kcal mol^{-1}. We re-calculated the product percentages with the new energy values.

Table 5.2. Benchmarking – inclusion of solvent models: Calculated percentage (TST) results are derived based on the TST assumption. The selectivity of the reaction entirely depends on the kinetics of TS1 and TS2. Calculated percentage (with VRAI) results incorporate the VRAI-selectivity calculation outcomes. We assume that the stereochemistry is controlled by kinetics via TS1 and the chemoselectivity, *ie* processes beyond TS1, are controlled by reaction dynamics. Unless otherwise specified, the level of theory for the calculations is ωB97XD/6-311++g(d,p)/SMD/toluene //B3LYP-D3/6-31g(d). The VRAI-selectivity calculations are based on frequency calculation at the B3LYP-D3/6-31g(d) level of theory.

	**Experimental Percentage**	**Calculated Percentage (TST)**	**Calculated Percentage (with VRAI)**
	Ketone-selective reaction - Cat-B [(a)]		
Aldehyde	0.00%	45.98%	5.12%
Epoxide	33.30%	1.50%	9.19%
Ketone (*R*)	44.70%	24.43%	57.62%
Ketone (*S*)	22.00%	28.08%	28.06%
	Ketone-selective reaction - Cat-C [(a)]		
Aldehyde	0.00%	21.10%	14.83%
Epoxide	25.00%	0.00%	23.27%
Ketone (*R*)	12.00%	0.00%	4.17%
Ketone (*S*)	63.00%	78.90%	57.73%
	Ketone-selective reaction - Cat-D [(a)]		
Aldehyde	0.00%	9.39%	8.25%
Epoxide	11.11%	0.00%	5.60%
Ketone (*R*)	3.56%	0.00%	2.80%
Ketone (*S*)	85.33%	90.61%	79.90%
	Epoxide-selective reaction - Cat-E [(b)]		
Aldehyde	(40.91%)	69.32%	44.16%
Epoxide (*SR*)	56.00%	9.93%	43.47%
Epoxide (*RS*)	0.28%	0.00%	0.00%
Epoxide (*SS* + *RR*)	2.81%	20.75%	3.37%
Ketone	0.00%	0.00%	9.00%
	Aldehyde-selective reaction - Cat-F<br>ωB97XD/6-311++g(d,p)/SMD/toluene //B3LYP-D3/6-31g(d)*		
Aldehyde (*S*)	1.04%	1.52%	NA
Aldehyde (*R*)	68.95%	0.00%	NA
Epoxide	0.00%	0.00%	NA
Ketone	30.00%	98.48%	NA
	ωB97XD/6-311++g(d,p)/PCM/toluene//B3LYP-D3/6-31g(d)		
Aldehyde (*S*)	1.04%	0.00%	0.00%
Aldehyde (*R*)	68.95%	5.14%	54.33%
Epoxide	0.00%	0.00%	0.00%
Ketone	30.00%	94.86%	45.67%

*In the energy check for the VRAI-selectivity test on TS0 as the first transition state, the second transition state (*ie* TS1) is higher in energy by 0.9 kcal mol^{-1} than the first transition state. In this scenario, the program would proceed to calculate the product percentages with the transition state theory (TST)

Table 5.3. Benchmarking – inclusion of solvent models: This table presents the mean absolute error (MAE) data of the calculated percentages compared to the experimental percentages. Unless otherwise specified, the level of theory for the calculations is ωB97XD/6-311++g(d,p)/SMD/toluene //B3LYP-D3/6-31g(d).

**MAE compared to the experimental percentage**	
Calculated Percentage (with VRAI)	Calculated Percentage (TST)

Ketone-selective reaction - Cat-B	
12.1%	26.0%
Ketone-selective reaction - Cat-C	
7.4%	18.5%
Ketone-selective reaction - Cat-D	
5.0%	7.3%
Epoxide-selective reaction - Cat-E	
4.2%	18.5%
Aldehyde-selective reaction - Cat-F ωB97XD/6-311++g(d,p)/PCM/toluene//B3LYP-D3/6-31g(d)	
7.8%	32.4%

Table 5.4. Benchmarking – inclusion of solvent models – ΔG values related to the dynamically controlled steps: ΔG from TS1 to INT2 (ΔG(TS1→INT2)) and ΔG from INT2 to TS2 (ΔG‡(TS2)) of the major pathways are recorded below for each reaction. The major pathway is the pathway with the highest %(Pathway). Unless otherwise specified, the level of theory for the calculations is ωB97XD/6-311++g(d,p)/SMD/toluene //B3LYP-D3/6-31g(d).

**ΔG(TS1→INT2)**	**ΔG‡(TS2)**
Ketone-selective reaction - Cat-B	
-9.7	2.5
Ketone-selective reaction - Cat-C	
-10.2	3.3
Ketone-selective reaction - Cat-D	
-10.1	3.9
Epoxide-selective reaction - Cat-E	
-12.1	0.4
Aldehyde-selective reaction - Cat-F ωB97XD/6-311++g(d,p)/PCM/toluene//B3LYP-D3/6-31g(d)	
-5.3	1.3

C. Structure re-optimisations

Key TS1, TS2 and INT2 structures with an $\Delta\Delta G^{\ddagger}$ < 2.5 kcal mol^{-1} at the ωB97XD/6-311g(d,p)//B3LYP-D3/6-31g(d) theory level were re-optimized at other levels of theory. The product percentages were calculated with the new energy values.

Table 5.5. Benchmarking – structure re-optimizations. Calculated percentage (TST) results are derived based on the TST assumption. The selectivity of the reaction entirely depends on the kinetics of TS1 and TS2. Calculated percentage (with VRAI) results incorporate the VRAI-selectivity calculation outcomes. We assume that the stereochemistry is controlled by kinetics via TS1 and the chemoselectivity, *ie* processes beyond TS1, are controlled by reaction dynamics.

	**Experimental Percentage**	**Calculated Percentage (TST)**	**Calculated Percentage (with VRAI)**
	ωB97XD/6-311g(d,p)//ωB97XD/6-31g(d)		
Aldehyde	0.00%	41.80%	40.60%
Epoxide	33.30%	5.80%	15.90%
Ketone (*R*)	44.70%	10.30%	10.80%
Ketone (*S*)	22.00%	42.10%	32.80%
	M06-2X/6-311g(d,p)//M06-2X/6-31G(d)		
Aldehyde	0.00%	80.10%	7.40%
Epoxide	33.30%	1.00%	4.80%
Ketone (*R*)	44.70%	3.70%	59.60%
Ketone (*S*)	22.00%	15.10%	28.20%
	ωb97xd/6-311++g(d,p)//CAM-B3LYP/6-31G(d)		
Aldehyde	0.00%	75.25%	1.86%
Epoxide	33.30%	7.87%	61.57%
Ketone (*R*)	44.70%	14.26%	35.63%
Ketone (*S*)	22.00%	2.62%	0.93%

Table 5.6. Benchmarking – structure re-optimizations: This table presents the mean absolute error (MAE) data of the calculated percentages compared to the experimental percentages.

**MAE compared to the experimental percentage**	
Calculated Percentage (with VRAI)	Calculated Percentage (TST)
ωB97XD/6-311g(d,p)//ωB97XD/6-31g(d)	
26%	31%
M06-2X/6-311g(d,p)//M06-2X/6-31G(d)	
14%	40%
ωb97xd/6-311++g(d,p)//CAM-B3LYP/6-31G(d)	
15%	38%

6. Quasi-classical molecular dynamics

A. Molecular dynamic simulations

Quasi-classical molecular dynamics (MD) simulations were conducted using Jprogdyn[10] in conjunction with Gaussian. The input structure for the MD simulation was the lowest energy TS1 of the ketone-selectivity reaction with Cat B. This is the simplest reaction system that we have invested in this study. A trial run was conducted with a timeframe from -500 fs to 500 fs. The default setting was employed (*ie* timestep = 1 fs). The result confirmed that the backward direction from the TS1 leads to the formation of the INT2 adduct.

An additional set of 10 trials was performed. Each trial commenced from the chosen TS1, with trajectories going in the backward direction. The MD calculations were terminated either upon the generation of a product or in the event of recrossing, characterized by the distance between forming C-C bonds exceeding 3.4 Å. Details of the calculation are in the table below.

Table 6.1. Results of the MD simulations

Run	Outcome	Timeframe of the simulation (fs)
0	Epoxide	555
1	Recrossing	1840
2	Recrossing	825
3	Epoxide	4855
4	Epoxide	1325
5	Recrossing	865
6	Epoxide	2250
7	Epoxide	2210
8	Epoxide	465
9	Epoxide	5330

B. Calculation time comparisons

With the lowest energy TS1 of the ketone-selectivity reaction with Cat B as an example, we compare the CPU time required to run the corresponding VRAI-selectivity calculation and the MD simulation for producing reaction trajectories.

VRAI-selectivity:

- The VRAI-selectivity calculation: 3.9 seconds on MacBook Pro (2 GHz Quad-Core Intel Core i5); CPU time (scaled) = 3.1

MD:

- It is worth emphasizing the computationally intensive nature of the MD simulation conducted. For the selected TS1, running a reaction trajectory spanning 400 fs requires 12 hours using 64 processors with icelake nodes (Intel Xeon scalable processors) on Cambridge CSD3 high-performance computing environment.
- The average timeframe for running one trajectory based on Table 6.1: 2052 fs
- Average CPU time (scaled) for running ten trajectories = 141 834 24

Producing ten MD trajectories vs the VRAI-selectivity calculation:
141 834 24 / 3.1 = 4 575 298

7. Notes on pervious works

In previous works, we developed and tested VRAI-selectivity on more than 60 reactions from the literatures.[6,11] Figure 7.1 presents a histogram that illustrate the distribution of the size of the chemical systems covered in our previous work.

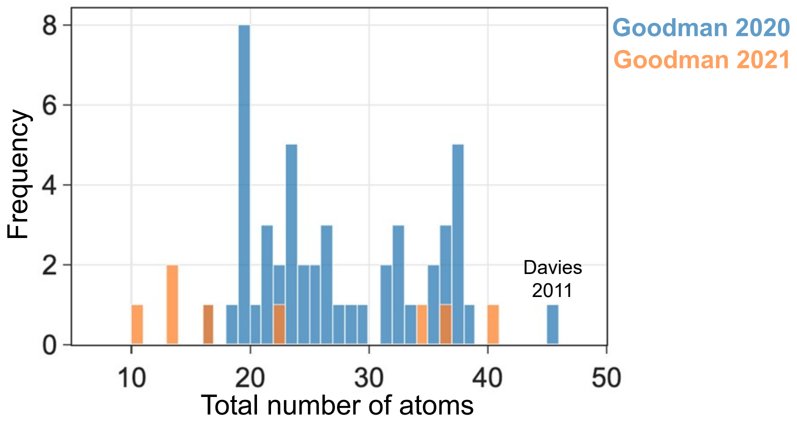

Figure 7.1. Distribution of the size of the chemical systems covered in our previous work.[6,11] The reaction with the greatest number of atoms comes from the work of Davies *et al.*[12]

8. Key Structures

The structural information is available in the Cambridge Apollo Repository:
https://doi.org/10.17863/CAM.96901

The key structures are included in the 'SI_key_structure_cobi' folder as opt+freq or opt Gaussian calculation output files. The optimisations were conducted at the B3LYP-D3/6-31G(d) level of theory. For the TSs, the filename listed below can be mapped to the label given in the calculation breakdown tables in Section 3 of this document.

```
SI_key_structure_cobi/
├─ .DS_Store
├─ aldehyde_selective/
│   ├─ INT/
│   │   ├─ jacs13ryu2_cat_2.out
│   │   ├─ jacs13ryu2_d_int1_R_1_TS0_diazo_qrc1.out
│   │   ├─ jacs13ryu2_d_int1_R_2.out
│   │   ├─ jacs13ryu2_d_int1_S_1.out
│   │   ├─ jacs13ryu2_d_int2_SRR_47.out
│   │   ├─ jacs13ryu2_d_int2_SRS_14.out
│   │   ├─ jacs13ryu2_d_int2_SSR_2.out
│   │   ├─ jacs13ryu2_d_int2_SSR_5_TS1_int0.out
│   │   └─ jacs13ryu2_d_int2_SSS_13.out
│   ├─ Product/
│   │   ├─ jacs13ryu2_d_int2_SRR_C_1_TSe_qrc2_3.out
│   │   ├─ jacs13ryu2_d_int2_SRR_H_1_TSe_qrc1_2.out
│   │   ├─ jacs13ryu2_d_int2_SRS_C_1_TSe_qrc2_2.out
│   │   └─ jacs13ryu2_d_int2_SRS_H_1_TSe_qrc1.out
│   ├─ TS0/
│   │   └─ jacs13ryu2_d_int1_R_1_TS0_diazo.out
│   ├─ TS1/
│   │   ├─ jacs13ryu2_d_int2_SRR_1_TS1_2.out
│   │   ├─ jacs13ryu2_d_int2_SRR_3_TS1.out
│   │   ├─ jacs13ryu2_d_int2_SRR_5_TS1.out
│   │   ├─ jacs13ryu2_d_int2_SRS_1_TS1.out
│   │   ├─ jacs13ryu2_d_int2_SRS_2_TS1.out
│   │   ├─ jacs13ryu2_d_int2_SRS_3_TS1.out
│   │   └─ jacs13ryu2_d_int2_SRS_4_TS1.out
│   └─ TS2/
│       ├─ jacs13ryu2_d_int2_SRR_C_1_TSe.out
│       ├─ jacs13ryu2_d_int2_SRR_H_1_TSe.out
│       ├─ jacs13ryu2_d_int2_SRR_H_3_TSe.out
│       ├─ jacs13ryu2_d_int2_SRR_O_4_TSe.out
│       ├─ jacs13ryu2_d_int2_SRS_C_1_TSe.out
│       ├─ jacs13ryu2_d_int2_SRS_C_2_TSe.out
│       ├─ jacs13ryu2_d_int2_SRS_C_3_TSe.out
│       ├─ jacs13ryu2_d_int2_SRS_C_4_TSe.out
│       ├─ jacs13ryu2_d_int2_SRS_H_1_TSe.out
│       ├─ jacs13ryu2_d_int2_SRS_H_2_TSe.out
│       └─ jacs13ryu2_d_int2_SRS_O_1_TSe.out
├─ benchmark/
│   ├─ CAM-B3LYP/
│   │   ├─ INT2/
│   │   │   ├─ ol17ryu_b_int2_SRR_19_cam.out
│   │   │   └─ ol17ryu_b_int2_SRS_1_cam.out
│   │   ├─ TS1/
│   │   │   ├─ ol17ryu_b_int2_SRR_2_TS1_cam.out
│   │   │   ├─ ol17ryu_b_int2_SRR_8_TS1_cam.out
│   │   │   ├─ ol17ryu_b_int2_SRS_1_TS1_cam.out
│   │   │   └─ ol17ryu_b_int2_SRS_9_TS1_cam.out
│   │   │
```

```
│       │       └─ TS2/
│       │           ├─ ol17ryu_b_int2_SRR_C_1_TSe_cam.out
│       │           ├─ ol17ryu_b_int2_SRR_C_2_TSe_cam.out
│       │           ├─ ol17ryu_b_int2_SRR_C_3_TSe_cam.out
│       │           ├─ ol17ryu_b_int2_SRR_H_1_TSe_cam.out
│       │           ├─ ol17ryu_b_int2_SRR_H_2_TSe_cam.out
│       │           ├─ ol17ryu_b_int2_SRS_C_1_TSe_cam.out
│       │           ├─ ol17ryu_b_int2_SRS_C_2_TSe_cam.out
│       │           ├─ ol17ryu_b_int2_SRS_C_3_TSe_cam.out
│       │           ├─ ol17ryu_b_int2_SRS_C_4_TSe_cam.out
│       │           ├─ ol17ryu_b_int2_SRS_C_5_TSe_cam.out
│       │           ├─ ol17ryu_b_int2_SRS_C_6_TSe_cam.out
│       │           ├─ ol17ryu_b_int2_SRS_H_4_TSe_cam.out
│       │           ├─ ol17ryu_b_int2_SRS_O_1_TSe_cam.out
│       │           ├─ ol17ryu_b_int2_SRS_O_2_TSe_cam.out
│       │           └─ ol17ryu_b_int2_SRS_O_5_TSe_cam.out
│       ├─ M062X/
│       │   ├─ INT2/
│       │   │   ├─ ol17ryu_b_int2_SRR_19_m0.out
│       │   │   └─ ol17ryu_b_int2_SRS_26_m0.out
│       │   ├─ TS1/
│       │   │   ├─ ol17ryu_b_int2_SRR_2_TS1_m0.out
│       │   │   ├─ ol17ryu_b_int2_SRR_8_TS1_m0.out
│       │   │   ├─ ol17ryu_b_int2_SRS_1_TS1_m0.out
│       │   │   └─ ol17ryu_b_int2_SRS_9_TS1_m0.out
│       │   └─ TS2/
│       │       ├─ ol17ryu_b_int2_SRR_C_1_TSe_m0.out
│       │       ├─ ol17ryu_b_int2_SRR_C_2_TSe_m0.out
│       │       ├─ ol17ryu_b_int2_SRR_H_1_TSe_m0.out
│       │       ├─ ol17ryu_b_int2_SRR_H_2_TSe_m0.out
│       │       ├─ ol17ryu_b_int2_SRS_C_1_TSe_m0.out
│       │       ├─ ol17ryu_b_int2_SRS_C_2_TSe_m0.out
│       │       ├─ ol17ryu_b_int2_SRS_C_3_TSe_m0.out
│       │       ├─ ol17ryu_b_int2_SRS_H_1_TSe_m0.out
│       │       ├─ ol17ryu_b_int2_SRS_H_2_TSe_m0.out
│       │       ├─ ol17ryu_b_int2_SRS_H_3_TSe_m0.out
│       │       ├─ ol17ryu_b_int2_SRS_H_4_TSe_m0.out
│       │       ├─ ol17ryu_b_int2_SRS_O_1_TSe_m0.out
│       │       ├─ ol17ryu_b_int2_SRS_O_2_TSe_m0.out
│       │       └─ ol17ryu_b_int2_SRS_O_5_TSe_m0.out
│       └─ wb97xd/
│           ├─ INT2/
│           │   ├─ ol17ryu_b_int2_SRR_19_wb.out
│           │   └─ ol17ryu_b_int2_SRS_26_wb.out
│           ├─ TS1/
│           │   ├─ ol17ryu_b_int2_SRR_2_TS1_wb.out
│           │   ├─ ol17ryu_b_int2_SRR_8_TS1_wb.out
│           │   ├─ ol17ryu_b_int2_SRS_1_TS1_wb.out
│           │   ├─ ol17ryu_b_int2_SRS_9_TS1_wb.out
│           │   ├─ ol17ryu_b_int2_SSR_3_TS1_wb.out
│           │   └─ ol17ryu_b_int2_SSS_c13_TS1_wb.out
│           └─ TS2/
│               ├─ ol17ryu_b_int2_SRR_C_1_TSe_wb.out
│               ├─ ol17ryu_b_int2_SRR_C_2_TSe_wb.out
│               ├─ ol17ryu_b_int2_SRR_C_3_TSe_wb.out
│               ├─ ol17ryu_b_int2_SRR_H_1_TSe_wb.out
│               ├─ ol17ryu_b_int2_SRR_H_2_TSe_wb.out
│               ├─ ol17ryu_b_int2_SRS_C_1_TSe_wb.out
│               ├─ ol17ryu_b_int2_SRS_C_2_TSe_wb.out
│               ├─ ol17ryu_b_int2_SRS_C_3_TSe_wb.out
│               ├─ ol17ryu_b_int2_SRS_H_1_TSe_wb.out
```

```
│           ├─ ol17ryu_b_int2_SRS_H_2_TSe_wb.out
│           ├─ ol17ryu_b_int2_SRS_H_3_TSe_wb.out
│           ├─ ol17ryu_b_int2_SRS_H_4_TSe_wb.out
│           ├─ ol17ryu_b_int2_SRS_O_1_TSe_wb.out
│           ├─ ol17ryu_b_int2_SRS_O_2_TSe_wb.out
│           └─ ol17ryu_b_int2_SRS_O_5_TSe_wb.out
├─ epoxide_selective/
│   ├─ INT/
│   │   ├─ agw21ryu_d_int1_R_2_2.out
│   │   ├─ agw21ryu_d_int1_S_5.out
│   │   ├─ agw21ryu_d_int2_SRR_2_2.out
│   │   ├─ agw21ryu_d_int2_SRS_8.out
│   │   ├─ agw21ryu_d_int2_SSR_22.out
│   │   ├─ agw21ryu_d_int2_SSS_74_2.out
│   │   ├─ agw21ryu_d_int2_SSS_Ca_1_TSe_qrc2_freq_int3.out
│   │   ├─ agw21ryu_d_int2_SSS_Ha_2_TSe_qrc1_freq_int3.out
│   │   └─ catd_2_3.out
│   ├─ Product/
│   │   ├─ agw21ryu_d_int2_SSS_Ca_1_H_TS2_qrc1.out
│   │   ├─ agw21ryu_d_int2_SSS_Ca_1_TS2_3_qrc2.out
│   │   ├─ agw21ryu_d_int2_SSS_Ha_2_C_TS3_2_qrc2.out
│   │   ├─ agw21ryu_d_int2_SSS_Ha_2_TS2_2_qrc1.out
│   │   ├─ agw21ryu_d_int2_SSS_Ha_3_TSe_qrc2.out
│   │   └─ agw21ryu_d_int2_SSS_Oa_2_TSe_qrc1.out
│   ├─ TS1/
│   │   ├─ agw21ryu_d_int2_SSR_2_TS1.out
│   │   ├─ agw21ryu_d_int2_SSR_3_TS1.out
│   │   ├─ agw21ryu_d_int2_SSS_10_TS1.out
│   │   ├─ agw21ryu_d_int2_SSS_15_TS1.out
│   │   ├─ agw21ryu_d_int2_SSS_50_TS1.out
│   │   └─ agw21ryu_d_int2_SSS_65_TS1.out
│   ├─ TS2/
│   │   ├─ agw21ryu_d_int2_SSS_Ca_1_TSe.out
│   │   ├─ agw21ryu_d_int2_SSS_Ha_2_TSe.out
│   │   ├─ agw21ryu_d_int2_SSS_Ha_3_TSe.out
│   │   └─ agw21ryu_d_int2_SSS_Oa_2_TSe.out
│   └─ TS3/
│       ├─ agw21ryu_d_int2_SSS_Ca_1_H_TS2.out
│       ├─ agw21ryu_d_int2_SSS_Ca_1_TS2_3.out
│       ├─ agw21ryu_d_int2_SSS_Ha_2_C_TS2_5.out
│       └─ agw21ryu_d_int2_SSS_Ha_2_TS2_2.out
└─ ketone_selective/
    ├─ with_cat-B/
    │   ├─ INT/
    │   │   ├─ ol17ryu_b_int1_R_1.out
    │   │   ├─ ol17ryu_b_int1_S_1.out
    │   │   ├─ ol17ryu_b_int2_SRR_19.out
    │   │   ├─ ol17ryu_b_int2_SRS_26.out
    │   │   └─ ol17ryu_catb_1.out
    │   ├─ Product/
    │   │   ├─ ol17ryu_b_int2_SRR_C_1_TSe_product.out
    │   │   ├─ ol17ryu_b_int2_SRR_H_1_TSe_product.out
    │   │   ├─ ol17ryu_b_int2_SRS_C_1_TSe_product.out
    │   │   ├─ ol17ryu_b_int2_SRS_H_4_TSe_product.out
    │   │   └─ ol17ryu_b_int2_SRS_O_2_TSe_product.out
    │   ├─ TS1/
    │   │   ├─ ol17ryu_b_int2_SRR_2_TS1.out
    │   │   ├─ ol17ryu_b_int2_SRR_8_TS1.out
    │   │   ├─ ol17ryu_b_int2_SRS_1_TS1_2.out
    │   │   └─ ol17ryu_b_int2_SRS_9_TS1.out
    │   └─ TS2/
    │
```

```
│         ├─ ol17ryu_b_int2_SRR_C_1_TSe.out
│         ├─ ol17ryu_b_int2_SRR_C_2_TSe.out
│         ├─ ol17ryu_b_int2_SRR_C_3_TSe.out
│         ├─ ol17ryu_b_int2_SRR_H_1_TSe.out
│         ├─ ol17ryu_b_int2_SRR_H_2_TSe.out
│         ├─ ol17ryu_b_int2_SRS_C_1_TSe.out
│         ├─ ol17ryu_b_int2_SRS_C_2_TSe.out
│         ├─ ol17ryu_b_int2_SRS_C_3_TSe.out
│         ├─ ol17ryu_b_int2_SRS_H_1_TSe.out
│         ├─ ol17ryu_b_int2_SRS_H_2_TSe.out
│         ├─ ol17ryu_b_int2_SRS_H_3_TSe.out
│         ├─ ol17ryu_b_int2_SRS_H_4_TSe.out
│         ├─ ol17ryu_b_int2_SRS_O_1_TSe.out
│         ├─ ol17ryu_b_int2_SRS_O_2_TSe.out
│         └─ ol17ryu_b_int2_SRS_O_5_TSe.out
├─ with_cat-C/
│    ├─ .DS_Store
│    ├─ INT/
│    │    ├─ ol17ryu_c_int1_R_5.out
│    │    ├─ ol17ryu_c_int1_S_2_2.out
│    │    ├─ ol17ryu_c_int2_SSR_2.out
│    │    ├─ ol17ryu_c_int2_SSS_64.out
│    │    └─ ol17ryu_catc_2.out
│    ├─ Product/
│    │    ├─ ol17ryu_c_int2_SSR_C_2_TSe_qrc1.out
│    │    ├─ ol17ryu_c_int2_SSR_H_3_TSe_qrc2.out
│    │    ├─ ol17ryu_c_int2_SSR_O_product.out
│    │    ├─ ol17ryu_c_int2_SSS_C_3_TSe_qrc1_2.out
│    │    └─ ol17ryu_c_int2_SSS_H_1_TSe_qrc1.out
│    ├─ TS1/
│    │    ├─ ol17ryu_c_int2_SRR_8_TS1.out
│    │    ├─ ol17ryu_c_int2_SSR_9_TS1.out
│    │    ├─ ol17ryu_c_int2_SSR_e11_TS1.out
│    │    ├─ ol17ryu_c_int2_SSR_e13_TS1.out
│    │    ├─ ol17ryu_c_int2_SSR_e3_TS1.out
│    │    └─ ol17ryu_c_int2_SSS_13_TS1.out
│    └─ TS2/
│         ├─ ol17ryu_c_int2_SSR_C_2_TSe.out
│         ├─ ol17ryu_c_int2_SSR_C_3_TSe.out
│         ├─ ol17ryu_c_int2_SSR_C_4_TSe.out
│         ├─ ol17ryu_c_int2_SSR_C_5_TSe.out
│         ├─ ol17ryu_c_int2_SSR_C_6_TSe.out
│         ├─ ol17ryu_c_int2_SSR_C_7_TSe.out
│         ├─ ol17ryu_c_int2_SSR_H_10_TSe.out
│         ├─ ol17ryu_c_int2_SSR_H_12_TSe.out
│         ├─ ol17ryu_c_int2_SSR_H_3_TSe.out
│         ├─ ol17ryu_c_int2_SSR_H_8_TSe.out
│         ├─ ol17ryu_c_int2_SSR_H_9_TSe.out
│         ├─ ol17ryu_c_int2_SSR_O_8_TSe.out
│         ├─ ol17ryu_c_int2_SSS_C_2_TSe.out
│         └─ ol17ryu_c_int2_SSS_C_3_TSe.out
└─ with_cat-D/
     ├─ INT2/
     │    ├─ ol17ryu_cate_11.out
     │    ├─ ol17ryu_e_int1_R_6.out
     │    ├─ ol17ryu_e_int1_S_2_6.out
     │    ├─ ol17ryu_e_int2_SSR_6_2.out
     │    └─ ol17ryu_e_int2_SSS_41.out
     ├─ Product/
     │    ├─ ol17ryu_e_int2_SSR_C_1_TSe_qrc1.out
     │    ├─ ol17ryu_e_int2_SSR_H_2_TSe_qrc2.out
```

```
│   ├── ol17ryu_e_int2_SSR_O_1_TSe_qrc2_2.out
│   ├── ol17ryu_e_int2_SSS_C_3_TSe_qrc1.out
│   ├── ol17ryu_e_int2_SSS_H_3_TSe_qrc1_3.out
│   └── ol17ryu_e_int2_SSS_O_1_TSe_qrc1.out
├── TS1/
│   ├── ol17ryu_e_int2_SSR_11_TS1.out
│   ├── ol17ryu_e_int2_SSR_13_TS1.out
│   ├── ol17ryu_e_int2_SSR_3_TS1.out
│   ├── ol17ryu_e_int2_SSR_9_TS1.out
│   ├── ol17ryu_e_int2_SSR_c9_TS1.out
│   ├── ol17ryu_e_int2_SSR_x1_TS1.out
│   ├── ol17ryu_e_int2_SSS_6_TS1.out
│   └── ol17ryu_e_int2_SSS_c13_TS1.out
└── TS2/
    ├── ol17ryu_e_int2_SSR_C_1_TSe.out
    ├── ol17ryu_e_int2_SSR_C_2_TSe.out
    ├── ol17ryu_e_int2_SSR_C_3_TSe.out
    ├── ol17ryu_e_int2_SSR_H_1_TSe.out
    ├── ol17ryu_e_int2_SSR_H_2_TSe.out
    ├── ol17ryu_e_int2_SSR_H_3_TSe.out
    ├── ol17ryu_e_int2_SSR_H_4_TSe.out
    ├── ol17ryu_e_int2_SSR_H_6_TSe.out
    ├── ol17ryu_e_int2_SSR_H_7_TSe.out
    ├── ol17ryu_e_int2_SSR_O_1_TSe.out
    ├── ol17ryu_e_int2_SSS_C_1_TSe.out
    ├── ol17ryu_e_int2_SSS_C_2_TSe.out
    ├── ol17ryu_e_int2_SSS_C_3_TSe.out
    ├── ol17ryu_e_int2_SSS_C_4_TSe.out
    ├── ol17ryu_e_int2_SSS_C_5_TSe.out
    ├── ol17ryu_e_int2_SSS_C_6_TSe.out
    ├── ol17ryu_e_int2_SSS_H_3_TSe.out
    └── ol17ryu_e_int2_SSS_O_1_TSe.out
```

9. Reference

1 J. M. Goodman and M. A. Silva, *Tetrahedron Lett.*, 2003, **44**, 8233–8236.

2 Schrödinger Release 2021-2 Maestro. Schrödinger, LLC, New York, NY, 2021.

3 C. Lu, C. Wu, D. Ghoreishi, W. Chen, L. Wang, W. Damm, G. A. Ross, M. K. Dahlgren, E. Russell, C. D. Von Bargen, R. Abel, R. A. Friesner and E. D. Harder, *J. Chem. Theory Comput.*, 2021, **17**, 4291–4300.

4 Collaborative Data Science. Plotly Technologies Inc. 2015. https://plot.ly (accessed June 02, 2023)

5 C. C. Lam and J. M. Goodman, *J. Chem. Inf. Model.*, 2023, **63**, 4364–4375.

6 S. Lee and J. M. Goodman, *Org. Biomol. Chem.*, 2021, **19**, 3940–3947.

7 S. Lee and J. M. Goodman, *J. Am. Chem. Soc.*, 2020, **142**, 9210–9219.

8 A. V Marenich, C. J. Cramer and D. G. Truhlar, *J. Phys. Chem. B*, 2009, **113**, 6378–6396.

9 G. Scalmani and M. J. Frisch, *J. Chem. Phys.*, 2010, **132**, 114110.

10 E. E. Kwan and R. Y. Liu, *J. Chem. Theory Comput.*, 2015, **11**, 5083–5089.

11 S. Lee and J. M. Goodman, *J. Am. Chem. Soc.*, 2020, **142**, 9210–9219.

12 J. H. Hansen, T. M. Gregg, S. R. Ovalles, Y. Lian, J. Autschbach and H. M. L. Davies, *J. Am. Chem. Soc.*, 2011, **133**, 5076–5085.
